# Inequality between women and men in ICD implantation

**DOI:** 10.1016/j.ijcha.2022.101075

**Published:** 2022-06-25

**Authors:** Sebastian Ingelaere, Ruben Hoffmann, Ipek Guler, Johan Vijgen, Georges H. Mairesse, Ivan Blankoff, Yves Vandekerckhove, Jean-Benoit le Polain de Waroux, Bert Vandenberk, Rik Willems

**Affiliations:** a University Hospitals Leuven, Department of Cardiovascular Diseases, Leuven, Belgium; b KU Leuven, Department of Cardiovascular Sciences, Leuven, Belgium; c KU Leuven, L-Biostat, Department of Public Health and Primary Care, Leuven, Belgium; d Jessa Ziekenhuis, Department of Cardiology, Hasselt, Belgium; e Cliniques du Sud Luxembourg, Department of Cardiology, Arlon, Belgium; f CHU Charleroi, Department of Cardiology, Charleroi, Belgium; g AZ Sint-Jan, Department of Cardiology, Brugge, Belgium; h University of Calgary, Libin Cardiovascular Institute, Calgary, Canada

**Keywords:** ICD implantation, Sex-differences, Clinical characteristics, Mortality

## Abstract

**Background:**

The impact of sex on ICD implantation practice and survival remain a topic of controversy. To assess sex-specific differences in ICD implantation practice we compared clinical characteristics and survival in women and men.

**Methods:**

From a nationwide registry, all new ICD implantations performed between 01/02/2010 and 31/01/2019 in Belgian patients were analyzed retrospectively. Baseline characteristics and survival rates were compared between sexes. To identify predictors of mortality, multivariable Cox regression was performed.

**Results:**

Only 3096 (20.9%) of 14,787 ICD implantations were performed in women. Within each type of underlying cardiomyopathy, the proportion women were lower than men. The main indication in men was ischemic vs dilated cardiomyopathy in women. Women were overall younger (59.1 ± 15.1 vs 62.6 ± 13.1 years; p < 0.001) and had less comorbidities except for oncological disease. More women functioned in NYHA-class III (33.6% vs 27.9%; p < 0.001) and had a QRS > 150 ms (29.4% vs 24.3%; p < 0.001), consistent with a higher use of CRT-D devices (31.7% vs 25.1%; p < 0.001). Women had more complications, reflected by the need to more re-interventions within 1 year (4.3% vs 2.7%, p < 0.001). After correction for covariates, sex-category was not a significant predictor of mortality (p = 0.055).

**Conclusion:**

There is a significant sex-disparity in ICD implantation rates, not fully explained by epidemiological differences in the prevalence of cardiomyopathies, which could imply an undertreatment of women. Women differ from men in baseline characteristics at implantation suggesting a selection bias. Further research is necessary to evaluate if women receive equal sudden cardiac death prevention.

## 1 Introduction

Annually, governmental statistics attribute 30 000 deaths to cardiovascular disease (CVD) in Belgium of which an important proportion suffers sudden cardiac death (SCD).[1] Ventricular arrhythmias (VA) are the main underlying mechanism of SCD and the implantation of an implantable cardioverter-defibrillator (ICD) is the most validated therapy to prevent arrhythmic death. [2] Patient selection is of utmost importance to optimize the cost-effectiveness of ICDs. Differences in treatment between men and women might influence outcome of women. In the field of cardiology, Dr. Bernadine Healy addressed this inequality in treatment for cardiovascular diseases in women and men as the “Yentl syndrome”, referring to a 19th century heroine Yentl, that disguised herself as a man to be allowed to attend school. [3] Indeed, research of Steingart et al. concluded that women with coronary heart disease were less likely to undergo coronary angiography, but if angiography was performed there was no significant difference in coronary bypass surgery rates. [4] This bias in the management of coronary heart disease led to the Go Red For Women (GRFW) campaigns by the American Heart Association and a call by the European Society of Cardiology to reduce the gap in the approach of cardiovascular disease between men and women after the recent publication of “the Lancet women and cardiovascular disease Commission: reducing the global burden by 2030”. [5] Sex-related inequalities in cardiac care is not isolated to coronary artery disease. Women were underrepresented in the landmark trials on ICD therapy and there is ongoing controversy regarding the benefit in women, with concerns about differences in complications and survival. [2], [5] To identify possible sex-specific differences in baseline characteristics and outcome in a real world setting, we conducted a retrospective study on all ICD implanted patients in Belgium.

## 2 Methods

### 2.1 Data source

The Belgian governmental health care institution and the Belgian Heart Rhythm Association keep track of all ICD device and ICD lead implantations or replacements in the Quality Electronic Registration of Medical acts, Implants and Devices (QERMID) registry. Participation by the 23 implanting centers is mandatory to obtain reimbursement of the materials used for the procedure. A database was extracted from this registry containing coded information on the specific procedure and patient. As parameter for complications, we used re-interventions within one year after primo ICD implantation that were registered in the QERMID registry, implying the use of new material, either device or lead, during this intervention. The primary endpoint of all-cause mortality data was obtained via the Crossroads Bank for Social Security of Belgium. A detailed description of the original registry and data processing can be found in the online supplement (Supplementary Appendix part I). The ethical committee of the University Hospitals of Leuven approved analyses on this retrospective database.

### 2.2 Study population

All patients with a first ICD implantation performed between February 1, 2010 and January 31, 2019 were eligible for inclusion. We excluded non-Belgian patients and patient with an unknown residency, because of missing data on their vital and socio-economic status.

### 2.3 Statistical analysis

We focused on the differences between women and men in baseline demographics and survival. We presented continuous variables as mean with standard deviation and categorical variables as number with percentage. After rejecting a normal distribution for age and left ventricular ejection fraction (LVEF) using the Kolmogorov-Smirnov test for normality, we compared continuous variables by a Mann-Whitney *U* test and categorical variables by a Chi^2^ test. After Bonferroni correction for multiple testing, only p-values ≤ 0.003 were considered significant. Secondly, evolution of implantations was assessed by a Chi^2^ test. Thirdly, we performed a Kaplan-Meier survival analysis with log-rank testing to compare women with men. To determine the predictors of mortality, we used Cox proportional hazard regression modelling. Variables with a p-value < 0.10 in univariable Cox analysis were entered in a multivariable regression model. We presented the hazard ratios (HR) with the according p-value. A HR greater than 1 indicates an increased mortality risk. To estimate the effect of sex on mortality and as a sensitivity test, three different propensity score methods (PSM) were used. We focus on the data using the nearest neighbor method without replacement, using common support and a caliper set at 0.0005. Two additional PSM were also explored and can be found in the online supplement part II. Statistical analyses were performed using SPSS (IBM Statistics, version 27, IBM Corp., Armonk, NY, USA), R software (R Foundation for Statistical Computing, version 3.6.2., Vienna, Austria) and STATA (StataCorp LCC, version 17, College Station, TX, USA).

## 3 Results

### 3.1 Baseline demographics

On 14,787 new ICD implantations, 3096 (20.9%) were performed in women (Table 1). While ischemic heart disease (IHD) was the main underlying heart disease in men (54.2% vs 27.9% in women; p < 0.001), non-ischemic heart disease (NIHD) was the most frequent pathology in women (44.8% vs 30.8% in men; p < 0.001). A higher proportion of women received an ICD because of arrhythmogenic heart disease (AHD) (23.1% vs 13.0% in men; p < 0.001) or adult congenital heart disease (ACHD) (0.6% vs 0.3% in men; p < 0.001). Within each subgroup of heart disease, women were a minority representing 12.0%, 27.8%, 32.0% and 36.7% of all IHD, NIHD, AHD and ACHD cases. There is a trend towards more ICD implantations in secondary prevention in women (34.1% vs 31.4% in men; p = 0.004, not significant after correction for multiple testing).

**Table 1:** Baseline patient characteristics.

		**Total**	**%**	**Men**	**%**	**Women**	**%**	**p-value**
N		14,787	100	11,691	79.1	3096	20.9	
**Age (mean ± sd)**								
		62.0 ± 13.6		62.6 ± 13.1		59.5 ± 15.1		**<0.001**
**LVEF (mean ± sd)**								
		35.0 ± 15.2		34.4 ± 14.7		37.2 ± 16.9		**<0.001**
**NYHA**	I	2324	15.7	1697	14.5	627	20.3	**<0.001**
	II	8132	55.0	6709	57.4	1423	46.0	
	III	4304	29.1	3264	27.9	1040	33.6	
	IV	27	0.2	21	0.2	6	0.2	
**Prevention**	Primary	10,059	68.0	8020	68.6	2039	65.9	0.004
	Secondary	4728	32.0	3671	31.4	1057	34.1	
**Heart Disease**	IHD	7197	48.7	6332	54.2	865	27.9	**<0.001**
	NIHD	4992	33.8	3606	30.8	1386	44.8	
	AHD	2229	15.1	1515	13.0	714	23.1	
	ACHD	49	0.3	31	0.3	18	0.6	
	Other	320	2.2	207	1.8	113	3.6	
**Type Device**	VVI/DDD	10,874	73.5	8759	74.9	2115	68.3	**<0.001**
	CRT-D	3913	26.5	2932	25.1	981	31.7	
**QRS**	<120	9704	65.6	7799	66.7	1905	61.5	**<0.001**
	120–150	1325	9.0	1043	8.9	282	9.1	
	150–180	2922	19.8	2155	18.4	767	24.8	
	>180	836	5.7	694	5.9	142	4.6	
**Re-interventions**	<1 y	444	3.0	312	2.7	132	4.3	**<0.001**
**AF**		3324	22.5	2777	23.8	547	17.7	**<0.001**
**Diabetes**		2209	14.9	1830	15.7	379	12.2	**<0.001**
**COPD**		1052	7.1	879	7.5	173	5.6	**<0.001**
**Neurological**		794	5.4	665	5.7	129	4.2	**<0.001**
**Oncological**		552	3.7	360	3.1	192	6.2	**<0.001**
**Renal Failure**		1475	10.0	1225	10.5	250	8.1	**<0.001**
**Center Volume**	high	9688	65.5	7643	65.4	2045	66.1	0.480
	low	5099	34.5	4048	34.6	1051	33.9	
**Population Density**	high	6905	46.7	5416	46.3	1489	48.1	0.184
	middle	6818	46.1	5421	46.4	1397	45.1	
	low	1064	7.2	854	7.3	210	6.8	
**Income**	high	4670	31.6	3684	31.5	986	31.8	0.725
	middle	6631	44.8	5262	45.0	1369	44.2	
	low	3486	23.6	2745	23.5	741	23.9	

Continuous variables expressed as mean ± SD and categorical variables as number with %. LVEF = Left Ventricular Ejection Fraction, NYHA = New York Heart Association classification of heart failure, AF = Atrial Fibrillation, COPD = Chronic Obstructive Pulmonary Disease. Center Volume with high vs low based on median. Population Density and Income divided in low (percentile 0–25), middle (percentile 25–75) and high (percentile 75–100). Accounting for an alpha of 0.05, only a p-value equal to or lower than 0.003 is considered significant after Bonferroni correction. P-values in bold reached statistical significance.

Overall, women were younger at implantation compared to men (59.5, SD 15.1 vs 62.6, SD 13.1 years; p < 0.001). However, there was no significant difference in age within the subgroups: IHD (65.8, SD 10.8 in women vs 66.3, SD 9.9 in men; p = 0.591), NIHD (61.7, SD 12.4 in women vs 62.2, SD 12.0 in men; p = 0.503), AHD (49.1, SD 17.4 in women vs 49.8, SD 17.0 in men; p = 0.387) nor ACHD (42.8, SD 19.7 in women vs 50.4, SD 18.2 in men; p = 0.168). Women had overall less comorbidities except for a history of oncological disease. Regarding socio-economic parameters, there were no significant differences in population density or average income of the area of residency between sexes. Furthermore, there was no difference in procedural volume of the implanting center nor clinical relevant discrepancies in sex-differences between high and low volume centers (Supplementary Appendix part II, table 1).

Most implantations involved a single or dual chamber device (VVI/DDD; 68.3% in women vs 74.9% in men; p < 0.001). Cardiac resynchronization therapy (CRT) was used in 31.7% of women, compared to 25.1% in men (p < 0.001). This is in line with a higher prevalence of heart failure symptoms and the presence of more broad QRS complexes in women (33.6% vs 27.9% NYHA III (p < 0.001) and 29.4% vs 24.3% QRS > 150 ms (p < 0.001) in women vs men).

### 3.2 Complications

Women experienced a higher re-intervention rate in the first year after implantation compared to men (4.3% vs 2.7%, p < 0.001) with especially more lead dislocations (1.5% vs 0.5%, p < 0.001) and perforations (0.2% vs 0.0%, p = 0.001). There was no difference regarding infections (0.5% vs 0.6%, p = 0.524) (Supplementary Appendix part II, table 2).

### 3.3 Mortality

**Fig. 1:**
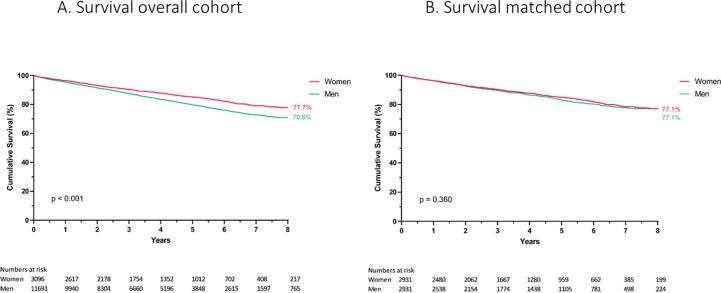
**Kaplan Meier survival curves. A.** Overall survival of ICD implanted patients stratified by sex category, log-rank p < 0.001. B. Survival of propensity score matched patients stratified by sex category.

**Fig. 2:**
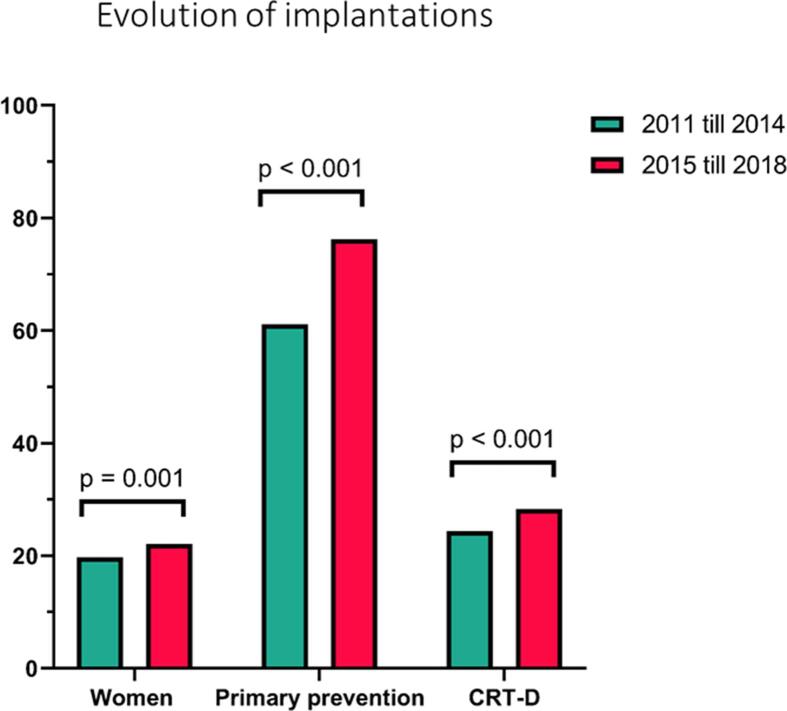
**Evolution of implantations.** Differences in implantation patterns between 2011–2014 and 2015–2018 for sex category, type of prevention and type of device. *Women:* 19.7 to 22.1%, p = 0.001; *Primary prevention:* 61.1 to 76.2%, p < 0.001; *CRT-D:* 24.4 to 28.3%, p < 0.001.

At time of closure of the study, we observed 2381 deaths (16.1%) with an average follow-up of 3.8 ± 2.5 years. In total, 12.5% of implanted women died compared to 17.1% of implanted men. For the years 2011 until 2017 (available years with at least 1 year of follow-up) 1-year mortality was stable and ranged between 4.0% (2013) and 5.0% (2015). Overall Kaplan-Meier survival analysis showed a significant better survival in women in general (log-rank p < 0.001) with an estimated mean survival of 7.86 years (95% CI 7.75–7.98) for women versus 7.48 years (95% CI 7.41–7.54) for men (Fig. 1A). This benefit remained significant when stratifying for type of prevention (primary vs secondary; log-rank p < 0.001) or device configuration (VVI/DDD vs CRT-D; log-rank p < 0.001) (Supplementary Appendix part II, Fig. 2). However, when stratifying for underlying heart disease, there was only a significant survival benefit for women with NIHD (Supplementary Appendix part II, figure 3). Univariable Cox regression analysis for the ICD population in general and after stratification by sex (Supplementary Appendix part II, table 3) withheld an unadjusted hazard ratio (HR) for mortality associated with male sex of 1.358 (95% CI 1.218–1.514; p < 0.001). However, after correction for covariates, male sex was no longer significantly associated with worse survival (adjusted HR of 1.116 (95% CI 0.997–1.248; p = 0.055)). This was confirmed by PSM as none of the three explored methods, could show a survival difference between sexes (Supplementary Appendix part II). Focusing on the matched cohort using the nearest neighbor method without replacement, survival curves did not diverge on the long term (Fig. 1B).

The multivariable Cox regression withheld age, secondary prevention indication, worse NYHA functional status, presence of atrial fibrillation (AF), presence of diabetes mellitus, presence of chronic obstructive pulmonary disease (COPD), presence of renal failure, history of neurological disease, history of oncological disease and low center volume as independent predictors of mortality (Table 2). A better-preserved LVEF was associated with a risk reduction as was implantation of a CRT-D device. When stratifying by sex category, we notice no gross differences in risk factors for mortality, except for the fact that the presence of COPD, history of oncological disease and center volume lost significance, but with comparable HR, possible due to the limited number of women in the analysis.

**Table 2:** Multivariable Cox Regression analysis for mortality.

		ALL (N = 14787)				MEN (N = 11691)				WOMEN (N = 3096)			
		HR	95% CI		p-value	HR	95% CI		p-value	HR	95% CI		p-value
**Sex**	F												
	M	1.116	*0.997*	*1.248*	0.055								
**Age**		1.051	*1.047*	*1.056*	**<0.001**	1.051	*1.046*	*1.057*	**<0.001**	1.051	*1.040*	*1.062*	<0.001
**LVEF**		0.981	*0.977*	*0.985*	**<0.001**	0.981	*0.976*	*0.985*	**<0.001**	0.981	*0.971*	*0.992*	<0.001
**NYHA**	I												
	II	1.306	*1.087*	*1.570*	0.004	1.252	*1.026*	*1.529*	0.027	1.600	*0.998*	*2.565*	0.051
	III	1.805	*1.461*	*2.231*	**<0.001**	1.742	*1.384*	*2.192*	**<0.001**	2.182	*1.271*	*3.746*	0.005
	IV	3.732	*1.948*	*7.151*	**<0.001**	3.130	*1.516*	*6.464*	0.002	8.980	*2.017*	*39.975*	0.004
**Prevention**	Primary												
	Secondary	1.402	*1.282*	*1.532*	**<0.001**	1.390	*1.262*	*1.531*	**<0.001**	1.509	*1.198*	*1.900*	<0.001
**Heart Disease**	Arrhythmogenic												
	Ischemic	1.033	*0.849*	*1.258*	0.743	1.040	*0.829*	*1.304*	0.734	1.046	*0.692*	*1.580*	0.832
	Non-ischemic	0.912	*0.744*	*1.117*	0.375	0.948	*0.750*	*1.199*	0.657	0.824	*0.540*	*1.258*	0.370
	Congenital	1.860	*0.870*	*3.976*	0.109	1.929	*0.785*	*4.738*	0.152	1.748	*0.415*	*7.364*	0.446
	Other	1.033	*0.658*	*1.620*	0.888	0.997	*0.579*	*1.717*	0.991	1.071	*0.478*	*2.399*	0.868
**Type of Device**	VVI/DDD												
	CRT-D	0.711	*0.601*	*0.842*	**<0.001**	0.743	*0.615*	*0.897*	0.002	0.598	*0.410*	*0.873*	0.008
**QRS (ms)**	<120												
	120–150	1.220	*1.027*	*1.448*	0.023	1.200	*0.993*	*1.450*	0.059	1.294	*0.861*	*1.945*	0.214
	150–180	1.086	*0.915*	*1.289*	0.343	1.077	*0.890*	*1.302*	0.446	1.135	*0.759*	*1.697*	0.537
	>180	0.940	*0.753*	*1.174*	0.587	0.895	*0.702*	*1.142*	0.374	1.185	*0.681*	*2.063*	0.548
**AF**		1.455	*1.332*	*1.588*	**<0.001**	1.438	*1.308*	*1.582*	**<0.001**	1.506	*1.200*	*1.889*	<0.001
**Diabetes**		1.337	*1.207*	*1.480*	**<0.001**	1.248	*1.116*	*1.395*	**<0.001**	1.928	*1.496*	*2.484*	<0.001
**COPD**		1.541	*1.359*	*1.749*	**<0.001**	1.580	*1.379*	*1.809*	**<0.001**	1.274	*0.896*	*1.811*	0.178
**Neurological**		1.168	*1.001*	*1.363*	0.048	1.104	*0.933*	*1.305*	0.249	1.646	*1.112*	*2.435*	0.013
**Oncological**		1.258	*1.047*	*1.511*	0.014	1.301	*1.057*	*1.603*	0.013	1.187	*0.803*	*1.755*	0.389
**Renal failure**		1.513	*1.351*	*1.694*	**<0.001**	1.453	*1.285*	*1.643*	**<0.001**	1.911	*1.431*	*2.551*	<0.001
**Center Volume**	high												
	low	1.200	*1.081*	*1.333*	0.001	1.245	*1.111*	*1.395*	**<0.001**	0.981	*0.750*	*1.283*	0.890
**Population density**	high												
	middle	0.916	*0.837*	*1.003*	0.057	0.907	*0.822*	*1.002*	0.054	0.932	*0.743*	*1.168*	0.539
	low	0.945	*0.796*	*1.122*	0.521	0.966	*0.803*	*1.163*	0.715	0.851	*0.539*	*1.343*	0.487
**Income**	high												
	middle	0.970	*0.877*	*1.073*	0.556	0.995	*0.891*	*1.111*	0.930	0.883	*0.682*	*1.143*	0.344
	low	1.117	*0.989*	*1.260*	0.074	1.090	*0.954*	*1.244*	0.206	1.388	*0.996*	*1.797*	0.053

Presentation of adjusted hazard ratios (HR) with their level of significance (p-value) for the whole group and stratified by sex category.

### 3.4 Evolution in time

When comparing implantations in the period between 2011 and 2014 with the period between 2015 and 2018, we noticed a small but significant increase in the proportion of women implanted with a new ICD device over time (19.7% vs 22.1%; p = 0.001). Moreover, there was a rise in implantations in primary prevention (61.1% vs 76.2%; p < 0.001) and CRT was used more often (24.4% vs 28.3%; p < 0.001) (Fig. 2).

## 4 Discussion

This retrospective analysis of a large nationwide cohort of patients implanted with an ICD demonstrated a pronounced difference in ICD implantation rate in women compared to men. Only a minority of ICD implantations occurred in women. As evident from their baseline characteristics, the selection of women for implantation was different. They had more dilated and arrhythmogenic cardiomyopathy and had less comorbidities at the time of implantation. Although they were overall younger, within each indication-category age was comparable between women and men. Differences in baseline characteristics could account for the better survival in women, since female sex itself was not a significant predictor of survival after correction for covariates and PSM.

### 4.1 Inequality between women and men

The low number of ICD implantations in women disclose sex disparities that may suggest undertreatment of women in Belgium.

#### 4.1.1 Inequality depending on underlying heart disease

To date, CVD is still the number one cause of death globally in men as well as women with the largest proportion being due to IHD. Within the European Union, data from the European Heart Network shows that IHD accounts for 14% of mortality in men and 12.0% in women. [6] The age-standardized prevalence of IHD in Belgium (anno 2015) was 1925/100 000 in men compared to 1032/100 000 in women. In 2017 the incidence of acute myocardial infarction (AMI) in Belgium is estimated to be 178/100 000 inhabitants of which 67.4% in men. [7] Therefore, it is astonishing that in the subgroup of IHD only 12% of implanted patients were women. In absolute numbers, this means a male to female implantation ratio of 7.3:1, compared to a 2:1 ratio of the prevalence of IHD or AMI. Sex-specific challenges in diagnosis of IHD might contribute to this gap, as women present more frequently with atypical symptoms and non-obstructive coronary artery disease. [5]

The treatment gap is less pronounced in the subgroup with NIHD in which the proportion of women was 27.8%. For the heterogeneous group of dilated cardiomyopathies (DCM), robust epidemiological data is lacking, with an estimated prevalence ranging from 1/2700 to 1/250 and conflicting data regarding sex ratios. [8] Even assuming a modest preponderance in males, undertreatment of women remains likely in NIHD given the 2.6:1 implantation ratio.

Except for long QT syndromes, with a higher arrhythmic risk in adult females compared to adult males, there is a higher disease penetrance and/or arrhythmic risk in men regarding Brugada syndrome, arrhythmogenic right ventricular cardiomyopathy and hypertrophic cardiomyopathy. [9] Thus, the lower proportion of women (32%) within this subgroup of AHD does not necessarily imply a sex inequality in ICD implantation rates.

Globally, there is an increasing prevalence of ACHD due to improved screening, medical and surgical treatment options during childhood. In a large population-based surveillance study in Atlanta (US), the male to female ratio was approximately 1:1 for all congenital lesions combined. However, there were large differences in the prevalence of specific congenital heart lesions with more diagnoses in boys of tetralogy of Fallot (TOF) and transposition of the great arteries (TGA), both lesions known to be at high risk for developing VA and SCD. [10] ACHD patients consist only a minority of the ICD implanted patients in our registry with the smallest difference in proportion (36.7% women vs 63.3% men) compared to the other subgroups.

#### 4.1.2 Inequality in primary vs secondary prevention

The use of an ICD was first addressed for secondary prevention in cardiac arrest survivors, who are considered to have a high risk of arrhythmic death. In our study, women represent 22.4% of all patients implanted with an ICD in secondary prevention. This is more compared to the average of 19.4% in the landmark trials (AVID, CASH and CIDS), but might be a consequence of different patient selection within our cohort with 43.4% of patients suffering from IHD compared to 69.0% in the trials. [11] Within our registry, we notice a discrete higher proportion of women with a secondary prevention indication (34.1% vs 31.4% in men; p = 0.004, not significant after correction for multiple testing).

Regarding primary prevention, the first landmark trials (MADIT-II and MUSTT) exclusively included IHD patients of which 17.6% were women, compared to 12.2% women with primary prevention ICD for IHD in our cohort. [12] After the DEFINITE (NIHD only) and SCD-HeFT (NIHD and IHD) trials including respectively 27.5% and 23.0% women, the indications for the use of prophylactic ICDs were broadened. [12] Considering NIHD patients with primary prevention indication in our dataset, 28.9% were women. Compared to the aforementioned landmark trials we implanted a similar percentage of women in primary prevention (19.4 vs 19.9% in trials).

In consonance with previous studies, the sex disparity in ICD implantation rates is less pronounced in secondary prevention (3.5:1) compared to primary prevention (3.9:1). While Curtis et al. found a less pronounced but existing treatment gap in secondary prevention, Johnson et al. states that the sex-specific treatment gap is driven by primary prevention indications alone. [13], [14] Interestingly, McLaughlin found that following aborted sudden cardiac death, women received less ICD therapy in general but not in a subgroup analysis of patients with shockable rhythms at presentation. [15]

#### 4.1.3 Inequality in type of device

In our cohort, women had more often a worse functional status and a QRS width > 150 ms explaining the significant higher proportion of women implanted with a CRT-D compared to men (31.7% vs 25.1%; p < 0.001]. This choice might be driven by the knowledge that women have been shown to respond better to CRT and at shorter QRS duration than in men. [5], [12], [16]

### 4.2 Is outcome really better in women?

#### 4.2.1 Complications

We report a higher complication rate in women in accordance with the existing literature. [17], [18], [19] After multivariable adjustment, female sex remains an independent predictor of major complications. [20], [21] An important caveat is that we only have information on complications with necessity of new ICD materials. In contrast with Lee et al. we found no impact of complications on survival, however comparison is impeded by differences in study design. [22]

#### 4.2.2 Mortality

There seemed to be a survival benefit in women. However, when adjusting for covariates in the Cox regression analysis or using PSM, sex category lost its significance as predictor of mortality. This suggests that differences in baseline characteristics - thus selection bias - rather than sex differences account for the difference in survival in the total cohort.

First, there was a higher proportion of women in the categories of heart disease with indication for ICD on younger age. Second, women had overall less comorbidities. Third, more women received a CRT-D with Woo et al. reporting a greater impact of resynchronization therapy in women, not seen in men. [16] Fourth, further exploration with subgroup analysis identified a sex-specific interaction between underlying cardiomyopathy and survival. Indeed, only in case of NIHD there was a significant benefit in women. At first glance, this is in contrast with the sex-specific analysis of the DEFINITE cohort by Albert et al. [23] However, the authors clearly state that the higher likelihood of non-cardiac death in ICD implanted women might represent a chance finding due to the limited population size. Indeed, the current available trials are underpowered to detect sex-by-treatment interactions. [24] Moreover, Adams et al. found a significant better survival for women with heart failure from NIHD compared to men with heart failure from any cause. [25] Literature on sex-related mortality differences in IHD are conflicting. [26] In our cohort, there is no difference in mortality between women and men with IHD. Patients with AHD had the best prognosis after ICD implantation with a similar survival in women and men.

In addition to multivariable Cox adjustment, we performed survival analysis on a PSM cohort, which could not withhold a survival difference between sexes. This difference between the non-corrected and corrected analyses favors the hypothesis that ICD implanted women are in general healthier than their male counterparts.

### 4.3 Limitations

Although we are working with real world data, there are several limitations. At first, we recognize the limitations of a retrospective study design. We acknowledge that there might be reporting bias and incomplete reporting by the implanting centers. Our analysis is limited to the available parameters of the registry, preventing direct comparison with large clinical trials or other registries. Especially the lack of information on medical treatment, cause of death, appropriate and inappropriate ICD interventions precludes thorough analysis.

We can only draw conclusions within ICD implanted patients, as we do not have a control cohort. We are well aware that our study lacks the denominator of patients eligible for ICD implantation that were not implanted. As such, we cannot proof actual referral or selection bias nor sex-specific differences in refusal rates. However, determining the true denominator would demand a longitudinal epidemiological cohort study, which would be logistically very challenging and by its nature would have an influence on the attitude of health care providers influencing potential bias. Therefore, we are limited to the comparison with epidemiological data and baseline characteristics as circumstantial evidence of a referral and selection bias.

### 4.4 Undertreatment of women?

Overall, less women were implanted with an ICD regardless the underlying cardiomyopathy, type of prevention or type of device. Although biological driven sex-differences in cardiovascular disease exert an influence, the current disparity compared to the epidemiological data suggests a possible undertreatment of women. The longstanding misperception that women have a negligible risk for SCD compared to men, may still be vivid. Therefore, physicians might perceive a differential benefit of ICD between women and men. The trend towards a higher proportion of secondary prevention compared to primary prevention indications in women might imply that women need to ‘prove’ their arrhythmic risk or need to be sicker before being implanted with an ICD.

Despite the lack of a denominator (all ICD eligible patients) to evaluate a true implantation deficit, our findings are consistent with previous observational studies. Indeed, a longitudinal study in Medicare beneficiaries by Curtis et al. withheld a lower implantation rate in women notwithstanding a history of cardiac arrest, the most unequivocal indication for ICD implantation. [13] Likewise, Hernandez et al. found that the overall use of ICD amongst eligible patients was low, with women being significantly less likely to receive an ICD compared to men. [27] Despite awareness campaigns and current guideline recommendations, this treatment gap persists in the modern era, especially in primary prevention. [14]

Another indirect argument in favor of actual undertreatment is the fact that women implanted with an ICD for IHD had the same age as their male counterparts, while IHD manifests later in life of women. At younger age, there is a pronounced gap in IHD prevalence, which disappears in octogenarians. Consequently, a higher mean age of women in the subgroup of IHD patients is to be expected. Advanced age might be, certainly in primary prevention, a hurdle for device implantation. [17]

On the other hand, more men suffer from heart failure with reduced ejection fraction (HFrEF) meeting the selection criteria in primary prevention more easily, while women are - in general - more affected by heart failure with preserved ejection fraction (HFpEF). [2], [5], [28] However, a study by Hess et al. showed that in patients admitted to the hospital with heart failure and LVEF < 35%, eligible for ICD implantation in primary prevention, women were counseled less frequently than men for ICD implantation. [29] Additionally, epidemiologic data by Gerber et al. with HFrEF defined as heart failure symptoms and a LVEF < 50% shows a male to female ratio in terms of incidence of 1.3:1 compared to an implantation rate of 4.2:1 in our dataset. [30] Ditto for the more recent study by Stolfo et al. with a cut-off of LVEF ≤ 40% to define HFrEF, there is a 2.5:1 ratio in incidence compared to a 4.2:1 ratio in implantation rate. [31]

#### 4.4.1 Comparison with other registries

Possible undertreatment of women is not unique for Belgium. In the SIMPLE trial, including patients from 18 different countries, approximately 19% of patients were women. [32] Annual reports from ICD registries from Germany and Spain facilitates comparison with our data. [33], [34] Furthermore the Swedish government keeps data on ICD implantations publicly available. [35] The proportion of female patients in the period 2011–2014 was 19.7% in our registry vs 21.6% in Germany, 18.2% in Spain and 19.8% in Sweden. More recently, from 2015 to 2018, we noticed a slight increase to 22.1% in our registry. Analogous there was an increase in Germany and Sweden to 21.9% and 20.4% respectively. Only in Spain a decrease to 17.0% occurred. There are no annual reports from the Dutch DIPR registry, nor from the French Stidefix registry and the UK NICOR report did not stratify by sex category.

#### 4.4.2 Is it getting better?

Increased awareness is an absolute first requirement to correct the imbalance in implantation rates and GRFW campaigns by the AHA might explain the increase in implantation rate in women over time. Furthermore, the IMPROVE-HF study showed that providing tools to support clinical decision-making can improve ICD implantation practice, regardless of sex category. [36] Since the DANISH trial, the debate about sense or nonsense of adding defibrillator therapy to CRT pacing has been heightened but did not affect the ICD implantation rates in our cohort. [37]

## 5 Conclusion

There is a significant disparity in ICD implantation rates between women and men, which cannot be explained purely by epidemiological differences in the prevalence of cardiomyopathies. Women differ significantly from men in baseline characteristics being younger and presenting with less comorbidities. This might imply selection bias and possible undertreatment of women, but the lack of a denominator limits a firm conclusion. Further research with focus on sex disparities is necessary to offer women and men an equal medical treatment.

## Disclosures

RW reports research funding from Abbott, Biotronik, Boston Scientific, Medtronic; speakers and consultancy fees from Medtronic, Boston Scientific, Biotronik, Abbott.

RW is supported as postdoctoral clinical researcher by the Fund for Scientific Research Flanders. BV is supported by a research grant of the Frans Van de Werf Fund for Clinical Cardiovascular Research.

## Declaration of Competing Interest

The authors declare that they have no known competing financial interests or personal relationships that could have appeared to influence the work reported in this paper.
