# Supplementary material for: Inequality between women and men in ICD implantation
Source: Int J Cardiol Heart Vasc. 2022 Jun 25;41:101075. doi: 10.1016/j.ijcha.2022.101075 (PMC9240366; doi:10.1016/j.ijcha.2022.101075)
Supplement: Supplementary data 1 [file mmc1.docx]

# Supplementary appendix:

1. Data extraction from national register

Database page 1

Data fields page 1

Categorization page 3

1. Additional statistical analyses

Complication rates page 16

Propensity score matching page 16

# Data extraction from national register

## DATABASE

All Implantable Cardioverter Defibrillator (ICD) procedures in Belgium are recorded in a digital register of the Belgian governmental health care institution (RIZIV/INAMI), the Quality Electronic Registration of Medical acts, Implants and Devices (QERMID) register. Data collection started 01/01/2010. Data about implantations between 01/07/2007 and 31/12/2009 were imported retrospectively into this register. Participation by implanting centers is mandatory to obtain reimbursement. Deadline for registration is 30 days after implantation.

The data from the register was extracted on 4/3/2019, for exploration by the Belgian Heart Rhythm Association (BeHRA), co-controller of the register. This database contains coded information on all ICD related procedures (new implants, ICD replacements and lead replacements) requiring new material with a total 29139 entries.

Duplicates (N=1038, 3.6%) were removed if they fulfilled 4 criteria: same patient code, same interaction date, same type of implantation and same indication.

All entries between 01/07/2007 and 1/02/2010 (N=4428, 15.2%) were excluded because of lack of mortality data during that period.

The remaining entries (N= 23 673, 81.2%) were divided into 3 main groups: primo implantations, re-implantations and other procedures. (figure 1)

## DATA FIELDS

The original database contained 264 data fields organized in columns with every column representing one data field and every row representing an entry (new implant, generator replacement or lead replacement). An overview of the data fields with a description can be found in table 1a.

To obtain a uniform database for further analysis we adjusted the dataset according to table 1b. This was necessary to limit the number of sub-classifications for analysis and to deal with historical changes in coding (categorization for indication *‘ConventionalIndication’* and QRS duration *‘QRSduration’)*.

Finally, we added new fields to the database based on the data within QERMID and other public available socio-economic databases: underlying pathology (*‘Pathology’*), group of heart disease (*‘HeartDisease’*), type of prevention (*‘Prevention*’), center volume (*‘CenterVolume’*), population density *(‘CategoryPopulation’*) and income (*‘CategoryIncome’*).

### Indications

The indication leading to the implantation is registered trough the data field ‘*ConventionalIndication*’ using a coded input. The coding changed per 1/1/2015, table 2 illustrated the algorithm used to account for these changes in the classification of the patients according to their underlying cardiomyopathy and primary or secondary prevention (see section on categorization).

### QRS duration

Data on QRS duration at implantation were entered as categorical variables (*‘QRSduration’)*. Categories changed over time (table 4). From 01/2010 till 07/2011, QRS-width broadening was divided into 4 categories, namely 120-140 ms, 141-160ms, 161-180ms and > 180ms. However, since 07/2011, cut-offs changed into 120-149ms, 150-180ms and > 180ms. Per 01/01/2015 an extra category for QRS-width < 120ms was added. We recoded this data field into *‘QRS’* with 4 categories: < 120ms, 120-150ms, 150-180ms, > 180ms. In case of absence of categorization, we presumed a QRS-width of < 120ms. The historical category 141-160ms was reclassified as 150-180ms.

### NYHA functional status

In the original database, information on the patients’ functional status according to the NYHA classification, is presented in *‘NYHAclass’* with 5 categories. We reclassified NYHA class IIa and IIb into NYHA class II to reduce the degrees of freedom with Chi² tests.

### Electrode configuration

We changed *‘ElectrodeConfiguration’* into *‘EC’* and reclassified RV lead and RA + RV lead into VVI/DDD and RA + RV + LV and RV + LV into CRT-D. Rationale is that different implantation patterns could be expected for conventional VVI/DDD devices versus resynchronization therapy.

### Atrial fibrillation

Information on atrial fibrillation can be found in two data fields. We recoded the data field *‘AtrialFibrillation’,* with dichotomous input, from “Y” or “N” into “1” or “0” in the data field *‘AF’.* The data field *‘AtrialFibrillationType’* was recoded into *‘AF_type’*, maintaining the categories paroxysmal, persistent and permanent atrial fibrillation, but with addition of “0” in case the patient is not known with atrial fibrillation.

### Prevention

The original database does not make a clear distinction between a primary or secondary prevention indication for the ICD implantation. Therefore, we added the data field *‘Prevention’* (see section on categorization). This facilitates comparison with landmark trials and other registries.

### Pathology

Using existing data fields we created a new field *‘Pathology’* to reclassify patients by underlying heart disease (see section on categorization) to allow comparisons between broader clinical entities.

### Center volume

Based on the average number of primo implantations per year per center in the period 2010-2019, we categorized the implanting centers as high or low volume. We used the median number of implantations per year of all centers as a cut-off. The centers implanted between 36 and 136 devices per year, with 47, 64 and 104 as respectively the p25, p50 (median) and p75.

### Socio-economic status

The ‘district’ where the patient resided, was available in the large majority of entries (N=23 250, 98.2% of included entries) as NIS code. The NIS coding system is a system used by the Belgian government and consists of 5 numbers. The first number indicates the province, the second number the arrondissement and the last 3 numbers the specific city. We added socio-economic information on the level of the arrondissement. Average income and population density are available per arrondissement from publicly available databases from the Belgian national institute for statistics (STATBEL). We used 2014 as the year of reference, as it is the median year of data entry into the database.

To define high, middle or low income areas we used the following cut-offs: >p75 for high income, p25-p75 for middle income and <p25 for low income areas. The same cut-offs were used to define high, middle or low populated areas.

## CATEGORIZATION

### Type of prevention

Based on indication and symptoms, we designed an algorithm to divide the indication for the ICD implantation into 3 groups: ‘primary prevention’, ‘secondary prevention due to reanimation’ and ‘secondary prevention due to syncope’. The algorithm takes into account differences in coding over time and is illustrated in table 2 and 3.

In detail:

- *‘ConventionalIndication’* group 1, corresponding with cardiac arrest, was assigned to the category ‘secondary prevention due to reanimation’, regardless the implantation date.

- For indication groups 2, 3.1 and 3.2, further categorization required additional input from the field *‘Symptoms’* (table 3). In case of symptom S1 – hypotension, S3 – presyncope or S4 – other, we assigned the patient as ‘primary prevention’. In case of symptom S2 – syncope, we assigned the patient to ‘secondary prevention due to syncope’.

- Indication group 4, used for implantations performed between 01/01/2010 and 31/12/2014, and groups 4.1 until 4.7 for implantations since 01/01/2015, were assigned to ‘secondary prevention due to syncope’.

- Indication groups 5, 6 and 7.1 until 7.4 were assigned to ‘primary prevention’, regardless of implantation date.

The majority (N=18 541, 78.3%) was classified automatically by this algorithm. Additionally, if one patient was classified correctly for the primary implantation but no data was available for the replacement, the same category of prevention was used (N=1 697, 7.2%). The remaining entries (N=3 435, 14.5%) were assigned manually by an expert (RW, SI) taking into account the free text data field *‘ExplanationIndication’.* This was feasible in N=1 361, 5.7%. The remaining unclassifiable entries were considered to be primary prevention (N= 2 074, 8.8%).

### Underlying Pathology

We reclassified patients by underlying cardiac disease into ischemic cardiomyopathy (ICMP), non-ischemic cardiomyopathy (NICM), hypertrophic cardiomyopathy (CMP_HCM), arrhythmogenic right ventricular cardiomyopathy (CMP_ARVC), long QT syndrome (CMP_LQT), Brugada syndrome (CMP_Brugada), sarcoidosis (CMP_sarcoidosis), other arrhythmogenic cardiomyopathies (CMP_other_arrhythmogenic) and congenital heart disease based on indication and aetiology or type of affection (table 6).

*‘ConventionalIndication’* groups 4.1 (since 01/01/2015), 7.1 and 7.2 correspond to ICMP. Groups 4.2 (since 01/01/2015), 7.3 and 7.4 to NICM. Groups 4.3, 4.4, 4.5, 4.6 and 4.7 correspond to CMP_LQT, CMP_Brugada, CMP_other_arrhythmogenic, CMP_HCM and CMP_ARVC respectively. For type of affection 5.1 long QT, 5.2 Brugada syndrome, 5.3 hypertrophic cardiomyopathy, 5.4 arrhythmogenic right ventricle dysplasia and 5.5 other conditions with an increased risk for ventricular arrhythmias, we assigned CMP_LQT, CMP_Brugada, CMP_HCM, CMP_ARVC and CMP_other_arrhythmogenic respectively. Group 6 corresponds with CMP_sarcoidosis.

We used additional information from the data fields ‘*Aetiology’* (table 5)*, ‘TypeAffection’* and *‘TypeCongenitalAffection’* to categorize groups 1, 2, 3.1 and 4. If aetiology described a history of myocardial infarction (C3-C10) we withheld ICMP. In case of coronaropathy without myocardial infarction (C11-C14), valvulopathy (G3) or dilated cardiomyopathie (G4) we assigned entries to NICM. For aetiologies hypertrophic cardiomyopathy (G5), dysplasia of the right ventricle (G6) and long QT (H2) categorization was possible towards CMP_HCM, CMP_ARVC and CMP_LQT. In case of a congenital heart disease, mentioned in the data field *‘TypeCongenitalAffection’,* we assigned the patients towards this category.

Entries with indication ‘3.2 – syncope without structural cardiac disease’ were exported (N=102, 0.4%) and reviewed by an expert (RW, SI) to classify them into a specific pathology, based on the free text data field *‘ExplanationIndication’*. In this way 46 entries or 45.1% of group 3.2 could be assigned to a specific pathology.

After this step, we performed a first check of the database for discordancy between the primary implantation and replacement procedure. If one patient was classified discordantly for the primary implantation vs replacement, the patient was assigned to the category of first implantation (N=1 311, 5.5%).

In a last step, the entries that were not assigned to a specific category of pathology (= ‘undef_pathology 99’) by the algorithm were exported (N=1 026, 4.3%) and reviewed by an expert (RW, SI) using the free text fields to classify them into a specific pathology if possible. This was possible only in 314 cases or 30.6%. After this, a second check was performed to avoid discordant labeling between first implantations and replacements and if necessary, patients were assigned to their first category (N=45, 0.2%).

### Heart disease

To reduce the degrees of freedom in statistical analysis, we grouped the underlying pathology in 5 general categories. The remaining categories are ischemic cardiomyopathy (= pathology group ICMP), non-ischemic cardiomyopathy (= pathology group NICM), arrhythmogenic cardiomyopathy (= pathology group CMP_HCM + CMP_ARVC + CMP_LQT + CMP_Brugada + CMP_sarcoidosis + CMP_other_arrhythmogenic), congenital cardiomyopathy (= pathology group congenital) and other (unspecified entries or pathology 99).


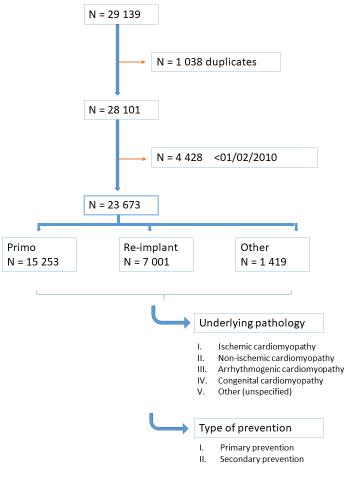


***Figure 1: General Flowchart***

| **NR** | **DATAFIELD** | **EXPLANATION** | **ADDITIONAL INFORMATION** | **APPLICABLE IF 'CONVENTIONAL INDICATION' IS** |
| --- | --- | --- | --- | --- |
| 1 | **ImplantCode** | Code linked to type of CIED | "101" = defibrillator |  |
| 2 | **Hospital agreement number** | Approval number of the hospital to implant ICDs |  |  |
| 3 | **PatientCode** | Coded patient ID |  |  |
| 4 | **SSN_Ind** | Indicates the status of synchronisation with CBSS | "Succes" or "NA" or "Failed" or "Mut" or "Initial" or "Unknown" |  |
| 5 | **Age** | Age at time of implantation |  |  |
| 6 | **Sex** | Sex of patient | "M" = male or "F" = female |  |
| 7 | **DateOfDeath_BCSS** | Date of death in CBSS |  |  |
| 8 | **District** | District of residency at time of implantation | NIS code |  |
| 9 | **Country** | Country of residency at time of implantation |  |  |
| 10 | **SpecialistCode** | Coded ID of responsible doctor |  |  |
| 11 | **RequestDate** | Date of request |  |  |
| 12 | **InteractionDate** | Date of implantation |  |  |
| * 13 | **TypeOfImplantation** | Type of implantation | "Primo" or "reimplant" or "addelectrode" or "addrepleelctrode" or "upgrade" |  |
| * 14 | **ConventionalIndication** | Code linked to the indication for implantation | Coding system by the RIZIV/INAMI *see table 2* |  |
| 15 | **DateCardiacArrest** | Date of cardiac arrest |  |  |
| 16 | **DateDocumentArrhythmia** | Date of documented arrhythmia |  |  |
| 17 | **DateSyncope** | Date of syncope |  |  |
| 18 | **DateElectrophExam** | Date of electrophysiological study |  |  |
| 19 | **DateCoronary** | Date of coronary angiography |  |  |
| 20 | **DateSpontaneousTachycardia** | Date of spontaneous tachycardia |  |  |
| 21 | **NonHospitalSetting** | Out of hospital | "Y"or "N" (yes or no) |  |
| 22 | **DescriptionCardiacArrest** | Additional information on the cardiac arrest |  |  |
| 23 | **TypeOfArrhythmia** | Type of arrhythmia |  |  |
| 24 | **Aetiology** | Underlying aetiology |  |  |
| 25 | **CommentAetiologyH3** | Additional information on aetiology |  |  |
| 26 | **InterventionalOrSurgicalHistoryA2** | Interventional or surgical history unknown | "Y"or "N" (yes or no) | 1 or 3.1 or 4 or 4.2 or 7.4 |
| 27 | **InterventionalOrSurgicalHistoryB1** | History of aorto-coronary bypass | "Y"or "N" (yes or no) | 1 or 3.1 or 4 or 4.2 or 7.4 |
| 28 | **InterventionalOrSurgicalHistoryB2** | Aneurysmectomy | "Y"or "N" (yes or no) | 1 or 3.1 or 4 or 4.2 or 7.4 |
| 29 | **InterventionalOrSurgicalHistoryB3** | Endocard resection | "Y"or "N" (yes or no) | 1 or 3.1 or 4 or 4.2 or 7.4 |
| 30 | **InterventionalOrSurgicalHistoryB4** | Cryosurgery | "Y"or "N" (yes or no) | 1 or 3.1 or 4 or 4.2 or 7.4 |
| 31 | **InterventionalOrSurgicalHistoryB5** | Other (surgery) | "Y"or "N" (yes or no) | 1 or 3.1 or 4 or 4.2 or 7.4 |
| 32 | **InterventionalOrSurgicalHistoryC1** | Direct current | "Y"or "N" (yes or no) | 1 or 3.1 or 4 or 4.2 or 7.4 |
| 33 | **InterventionalOrSurgicalHistoryC2** | Radiofrequency | "Y"or "N" (yes or no) | 1 or 3.1 or 4 or 4.2 or 7.4 |
| 34 | **InterventionalOrSurgicalHistoryC3** | Alcoholization | "Y"or "N" (yes or no) | 1 or 3.1 or 4 or 4.2 or 7.4 |
| 35 | **InterventionalOrSurgicalHistoryC4** | Other (catheter ablation) | "Y"or "N" (yes or no) | 1 or 3.1 or 4 or 4.2 or 7.4 |
| 36 | **InterventionalOrSurgicalHistoryD1** | Terminating tachycardia (pacing) | "Y"or "N" (yes or no) | 1 or 3.1 or 4 or 4.2 or 7.4 |
| 37 | **InterventionalOrSurgicalHistoryD2** | Other (pacing) | "Y"or "N" (yes or no) | 1 or 3.1 or 4 or 4.2 or 7.4 |
| 38 | **InterventionalOrSurgicalHistoryE1** | Other cardiac history | "Y"or "N" (yes or no) | 1 or 3.1 or 4 or 4.2 or 7.4 |
| 39 | **InterventionalOrSurgicalHistoryF1** | None of the above | "Y"or "N" (yes or no) | 1 or 3.1 or 4 or 4.2 or 7.4 |
| 40 | **CommentHistoryE1** | Additional information on E1 |  |  |
| 41 | **FrequencyTachycardia** | Frequency of tachycardia (bpm) |  |  |
| 42 | **Symptoms** | Code linked to symptoms | from 1/1/2010 till 31/12/2014 *see table 3* | 2 or 3.1 or 3.2 |
| 43 | **CommentSymptomS4** | Additional information on symptom 'S4' |  |  |
| 44 | **Waitinglist** | Patient activated on waiting list | "Y"or "N" (yes or no) |  |
| 45 | **CommentWaitinglistNO** | Motivation if "N" |  |  |
| * 46 | **TypeCongenitalAffection** | Type of congenital affection | 30051**01** = TOF, -**02** = septal defect, -**03** = TGA, -**04** = isolated congenital valvular disease, - **05** = DORV, - **06 =** Ebstein anomaly, -**07 =** Coarctatio Aortae, -**08 =** univentricular heart, -**09 =** other | 2 (since 1/1/2015) |
| 47 | **Cardioversion** | History of cardioversion | "Y"or "N" (yes or no) |  |
| 48 | **DateCardioversion** | If "Y": date of cardioversion |  |  |
| 49 | **ReasonNoCardioversion** | If "N": reason |  |  |
| 50 | **PreviousMedicalCare** | Previous care | 30029**01 =** medical, -**02** = ablation, -**03** = other, -**04** = none |  |
| 51 | **CommentCareT3** | If "T3": explantation |  |  |
| 52 | **MotivationDefibImplantation** | Motivation of defibrillator implantation |  |  |
| 53 | **DrugTherapy** | Use of drug therapy | 30053**01 =** beta-blocker, -**02 =** amiodarone, -**03 =** other | 4.3 or 4.4 or 4.5 or 4.6 or 4.7 |
| 54 | **DrugTherapyDescription** | Description of drug therapy if "other" |  | 4.3 or 4.4 or 4.5 or 4.6 or 4.7 |
| 55 | **FamilyAntecedents** | Family history | "Y"or "N" (yes or no) | 4.3 or 4.4 or 4.5 or 4.6 or 4.7 |
| 56 | **RiscScore** | Risk score |  | 4.6 or 5.3 |
| 57 | **TypeAffection** | Type of affection | 30027**01 =** LQTS, -**02 =** BrS, **-03 =** HCM, **-04 =** ARVC, **-05 =** other | 5 or 6 |
| 58 | **CommentAffection5.5** | Explanation if "other" |  | 5 |
| 59 | **RiskFactorsF1** | Electrocardiological anomaly | "Y"or "N" (yes or no) | 5 |
| 60 | **RiskFactorsF2** | Anatomical anomaly | "Y"or "N" (yes or no) | 5 |
| 61 | **RiskFactorsF3** | Result of electrofysiological exam | "Y"or "N" (yes or no) | 5 |
| 62 | **RiskFactorsF4** | Pharmacological test | "Y"or "N" (yes or no) | 5 |
| 63 | **RiskFactorsF5** | Exercise test | "Y"or "N" (yes or no) | 5 |
| 64 | **RiskFactorsF6** | Spontenaous arrhythmia without syncope | "Y"or "N" (yes or no) | 5 |
| 65 | **RiskFactorsF7** | Other arrhythmia (bradycardia, AV-block) | "Y"or "N" (yes or no) | 5 |
| 66 | **RiskFactorsF8** | Nature of genetic anomaly | "Y"or "N" (yes or no) | 5 |
| 67 | **RiskFactorsF9** | Associated myocardial ischemia | "Y"or "N" (yes or no) | 5 |
| 68 | **RiskFactorsF10** | Family history | "Y"or "N" (yes or no) | 5 |
| 69 | **RiskFactorsF11** | Other | "Y"or "N" (yes or no) | 5 |
| 70 | **CommentsRiskFactorF11** | Explanation if "other" |  | 5 |
| 71 | **ExplanationIndication** | Motivation for indication |  | 4.1 or 5 or 7.1 or 7.2 |
| 72 | **TypeInducedArrhythmia** | Type of induced arrhythmia | 30016**01 =** VF, -**02 =** monomorph VT, -**03 =** sustained polymorph VT | 6 or 7.2 |
| * 73 | **InfarctMoment** | Time since infarction |  | 4.1 or 7.1 or 7.2 |
| 74 | **RevascularisationType** | Revascularisation | 30015**01 =** None, **-02 =** Angioplasty, -**03 =** Surgically | 4.1 or 7.1 or 7.2 |
| 75 | **ExplanationRevascularNONE** | Explanation if "none" |  | 4.1 or 7.1 or 7.2 |
| 76 | **DateMyocardialRevascularisation** | Date of myocardial revascularisation |  | 4.1 or 7.1 or 7.2 |
| 77 | **RevascularisationSucces** | Succes of revascularisation | "Y"or "N" (yes or no) | 4.1 or 7.1 or 7.2 |
| 78 | **ExplanationResvascularNOSuccess** | Explanation in case of "N" |  | 4.1 or 7.1 or 7.2 |
| 79 | **PreviousDrugCare1** | Coded input for medical treatment | 30031**01** = diuretics, **-02** = beta-blockers, **-03** = ACE-inhibitors, -**04** = digitalis, -**05** = other | 7.3 |
| 80 | **PreviousDrugCare2** |  |  | 7.3 |
| 81 | **PreviousDrugCare3** |  |  | 7.3 |
| 82 | **PreviousDrugCare4** |  |  | 7.3 |
| 83 | **CommentDrugCareT5** | Explanation if "T5" |  | 7.3 |
| 84 | **TypeSyncope** | Coded input for syncope |  | 4.2 or 7.4 |
| 85 | **CommentSyncopeS5** | Additional information |  | 4.2 or 7.4 |
| 86 | **ArgumentsTachyarrhythmiaBaseSyncope** | Motivation to link tachy-arrhythmia to event of syncope | | 4.2 or 7.4 |
| 87 | **LVEFpercentage** | Left ventricular ejection fraction (%) | Mandatory field for PRIMO implantations or upgrade to resynchronisation |  |
| 88 | **Angiography** | LVEF by angiography | "Y"or "N" (yes or no) |  |
| 89 | **Scintigraphy** | LVEF by scintigraphy | "Y"or "N" (yes or no) |  |
| 90 | **Ultrasound** | LVEF by ultrasound | "Y"or "N" (yes or no) | 1 or 2 or 3.1 or 3.2, for 5 or 6 (till 31/12/2014) and upgrades |
| 91 | **CardiacMRI** | LVEF by cardiac MRI | "Y"or "N" (yes or no) |  |
| 92 | **NYHAclass** | Coded input for NYHA functional status | 30013**01** = class I, -**02** = class IIa, **-03** = class IIb, -**04** = class III, -**05** = class IV |  |
| 93 | **CommentsIndication** | General remarks on indication |  |  |
| 94 | **ImplantationDateExplantedDevice** | Implantation date of explanted device |  |  |
| 95 | **FirmExplantedDevice** | Firm of explanted device | 300(**01/44**)**01 =** Biotronik, -**02**  = Boston Scientific, **-03 =** Medtronic, **-04** = St-Jude Medical, **-05** = Sorin, 300**0106 =** LivaNova, 300**4406 =** Unknwon |  |
| 96 | **ModelExplantedDevice** | Model of explanted device |  |  |
| 97 | **IdCodeExplantedDevice** | ID code explanted device (if available) |  |  |
| 98 | **NomenclaturenbrDevice** | Nomenclature number of implanted device |  |  |
| 99 | **IDCodeDevice** | ID code of device |  |  |
| 100 | **FirmDevice** | Firm of device | 30001**01 =** Biotronik, -**02**  = Boston Scientific, **-03 =** Medtronic, **-04** = St-Jude Medical, **-05** = Sorin, -**06 =** LivaNova |  |
| 101 | **ModelDevice** | Model of device |  |  |
| 102 | **SerialnbrDevice** | Serial number of device |  |  |
| 103 | **ImplantationSite** | Site of implantation | "unknown", "sub-rectus", "supra-rectus", "intra thoracic", "pre-pectoral", "sub-pectoral", "axillary", "other" |  |
| 104 | **OtherSurgery** | Related surgical history | 30002**01 =** unknown, -**02** = aorto-coronary bypass, **-03 =** valve replacement, **-04 =** valvuloplasty, **-05 =** Other valve related procedure, **-06 =** endocardial resection, **-07 =** cryosurgery, **-08 =** aneurysmectomy, **-09 =** other arrhythmogenic, **-10 =** other general, **-11 =** none |  |
| 105 | **ElectrodeConfiguration** | Electrode configuration | 30036**01 =** RV, **-02 =** RA+RV, **-03 =** RA+RV+LV, **-04=** RV+LV |  |
| 106 | **ReasonReplacement** | Reason of replacement | 30007**01** = Unknown, **-02** = Elective, **-03 =** Recall, **-04 =** System change - elective problem, **-05 =** Upgrade to different system, **-06 =**  Other (elective), **-07 =**  Erosion, **-08 =** Infection, **-09 =** Wound pain, **-10 =** Other (surgical), **-11 =** Unspecified failure, **-12 =** Undersensing, **-13 =** Oversensing, **-14 =** Programming, **-15 =** No output, **-16 =** Low pacing defibrillator output, **-17 =** Slow pacing rate, **-18 =**  Runaway pacing rate, **-19 =** Connector failure, **-20 =** Encapsulation failure, **-21 =** Unnecessary shocks not related to sensing, **-22 =** Other (failure), **-23 =** Normal EOL, **-24 =** Premature EOL, **-25 =**  Other (failure battery), **-26 =** Other |  |
| 107 | **CommentsReasonG1** | Motivation of replacement if non of the coded indications is applicable | |  |
| 108 | **NbrOfshocks** | Number of shocks |  |  |
| 109 | **NbrTreatmentAntiTachycardiaPacing** | Number of treatments with anti-tachycardia pacing |  |  |
| 110 | **CommentsDevice** | General remarks on device |  |  |
| 111 | **DecisionDateDevice** | Decision date device |  |  |
| 112 | **DecisionDevice** | Decision regarding reimbursement of device |  |  |
| 113 | **ReasonRefusalDevice** | Motivation to refuse reimbursement |  |  |
| 114 | **CommentsDecisionDevice** | General remarks on decision for device |  |  |
| 115 | **Electrodetype1** | Type of the electrode | 30011**01 =** Unknown, **-02 =** Ventricular unipolar - epicardial (sense/pace only), **-03 =** Ventricular bipolar - epicardial (sense/pace only), **-04 =** Ventricular unipolar - endocardial (sense/pace only), **-05 =** Ventricular bipolar - endocardial (sense/pace only), **-06 =** Atrial unipolar - epicardial (sense/pace only), **-07 =** Atrial unipolar - endocardial (sense/pace only), **-08 =** Atrial bipolar - endocardial (sense/pace only), **-09 =** Other (détection/stimulation only), **-10 =** LV patch – epicardial, **-11 =** RV patch – epicardial, **-12 =** Other patch – epicardial, **-13 =**  Patch – subcutaneous, **-14 =** Array subcutaneous, -**15 =** RA/SVC coil – endocardial, **-16 =** Coronary sinus coil – endocardial, -**17 =** RV coil – endocardial, **-18 =** RV coil + RA/SVC coil – endocardial, **-19 =** Other |  |
| 116 | **NomenclaturenbrElectrode1** | Nomenclature number of the electrode |  |  |
| 117 | **IDCodeElectrode1** | ID code of the electrode |  |  |
| 118 | **NotificationcodeElectrode1** | Notification code of electrode |  |  |
| 119 | **ClassificationcodeElectrode1** | Classification code of electrode |  |  |
| 120 | **FirmElectrode1** | Firm of electrode | 30001**01** = Biotronik, -**02** = Boston Scientific, -**03** = Medtronic, -**04** = St-Jude Medical, -**05** = Sorin, -**06** = LivaNova **OR** 30047**01** = Be.Med, **-02 =** Biotronik, **-03 =** Boston Scientific, -**04 =** Medevico, **-05 =** Medtronic, **-06 =** St. Jude Medical, **-07 =** Sorin Group Belgium, **-08 =** Teleflex Medical, **-09 =** LivaNova |  |
| 121 | **ModelElectrode1** | Model of electrode |  |  |
| 122 | **SerialNbrElectrode1** | Serial number of electrode |  |  |
| 123 | **SubtypeIntervention1** | Subtype of intervention | "A" = addition, "R" = replacement, since 1/1/2015 |  |
| 124 | **ReasonReplacement1** | Reason to replace | 30008**01 =** Unknown, **-02 =**  Elective, **-03 =** Displacement, **-04 =** Pacing exit block, **-05 =** EMG activity, **-06 =** Extracardiac inhibition, **-07 =** Perforation, **-08 =**  Undersensing, **-09 =** High DFT, **-10 =**  Patch crumpling, **-11 =** Other (Elective), **-12 =** Infection, **-13 =** Erosion, **-14 =** Other (surgical), **-15 =** Connector failure, **-16 =** Insulation break, **-17 =** Conductor break, **-18 =** Other (failure) |  |
| 125 | **ReasonAddElectrode1** | Reason to add electrode | till 31/12/2014 |  |
| 126 | **CommentsElectrode1** | General remarks on electrode |  |  |
| 127 | **DecisionDateElectrode1** | Decision date electrode |  |  |
| 128 | **DecisionElectrode1** | Decidion regarding reimbursement of electrode |  |  |
| 129 | **ReasonRefusalElectrode1** | Motivation to refuse reimbursement |  |  |
| 130 | **CommentsDecisionElectrode1** | General remarks on decision for electrode |  |  |
|  |  | ***Field number 115 till 130 are repeated for up to 5 electrodes*** |  |  |
| 195 | **DurationIntervention** | Duration of intervention (minutes) |  |  |
| 196 | **RadiationDose** | Radiation dose | since 1/1/2015 |  |
| 197 | **Doctor implanter** | Qualification of implanting doctor(s) | 30000**15** = Cardiologist, -**16** = Surgeon, -**17** = Both |  |
| 198 | **Acute complicationsA1** | None | "Y"or "N" (yes or no) |  |
| 199 | **Acute complicationsB1** | Pneumothorax | "Y"or "N" (yes or no) |  |
| 200 | **Acute complicationsB2** | Haemothorax | "Y"or "N" (yes or no) |  |
| 201 | **Acute complicationsB3** | Thoracic outlet lesion | "Y"or "N" (yes or no) |  |
| 202 | **Acute complicationsB4** | Nerve plexus lesion | "Y"or "N" (yes or no) |  |
| 203 | **Acute complicationsB5** | Air embolism | "Y"or "N" (yes or no) |  |
| 204 | **Acute complicationsB6** | Venous perforation | "Y"or "N" (yes or no) |  |
| 205 | **Acute complicationsB7** | Venous thrombosis | "Y"or "N" (yes or no) |  |
| 206 | **Acute complicationsB8** | Arterial puncture | "Y"or "N" (yes or no) |  |
| 207 | **Acute complicationsB9** | Bleeding leading to transfusion | "Y"or "N" (yes or no) |  |
| 208 | **Acute complicationsC1** | Unexpected sustained AV-block | "Y"or "N" (yes or no) |  |
| 209 | **Acute complicationsC2** | Sustained supraventricular tachycardia | "Y"or "N" (yes or no) |  |
| 210 | **Acute complicationsC3** | Sustained atrial fibrillation | "Y"or "N" (yes or no) |  |
| 211 | **Acute complicationsC4** | Sustained atrial flutter | "Y"or "N" (yes or no) |  |
| 212 | **Acute complicationsC5** | Sustained ventricular tachycardia | "Y"or "N" (yes or no) |  |
| 213 | **Acute complicationsC6** | Ventricular fibrillation | "Y"or "N" (yes or no) |  |
| 214 | **Acute complicationsD1** | Atrial wall perforation | "Y"or "N" (yes or no) |  |
| 215 | **Acute complicationsD2** | Ventricular wall perforation | "Y"or "N" (yes or no) |  |
| 216 | **Acute complicationsD3** | Pericardial effusion | "Y"or "N" (yes or no) |  |
| 217 | **Acute complicationsD4** | Pericardial tamponade | "Y"or "N" (yes or no) |  |
| 218 | **Acute complicationsD5** | Valve lesion | "Y"or "N" (yes or no) |  |
| 219 | **Acute complicationsD6** | Dissection of the coronary sinus | "Y"or "N" (yes or no) |  |
| 220 | **Acute complicationsE1** | Failed connection device | "Y"or "N" (yes or no) |  |
| 221 | **Acute complicationsE2** | Dislocation ventricular electrode | "Y"or "N" (yes or no) |  |
| 222 | **Acute complicationsE3** | Dislocation atrial electrode | "Y"or "N" (yes or no) |  |
| 223 | **Acute complicationsF1** | Hematoma of significant size | "Y"or "N" (yes or no) |  |
| 224 | **Acute complicationsF2** | Loosening sutures | "Y"or "N" (yes or no) |  |
| 225 | **Acute complicationsG1** | Stimulation of the diapraghm | "Y"or "N" (yes or no) |  |
| 226 | **Acute complicationsG2** | Stimulation of the phrenic nerve | "Y"or "N" (yes or no) |  |
| 227 | **DualTripleChamberIndication** | Indication for dual or triple chamber device | 30025**01 =** Sinus bradycardia, **-02 =** AV conduction defect, **-03 =** atrial arrhyhthmia, **-04 =** slow VT, **-05 =** future candidate CRT-D |  |
| 228 | **ResynchronisationTherapyIndication** | Indication for resynchronisation present | "Y" or "N" (yes or no), till 31/12/2014 |  |
| 229 | **LBBB** | Presence of left bundle branch block (LBBB) | "Y" or "N" (yes or no) |  |
| * 230 | **QRSduration** | QRS duration | *see table 4* |  |
| 231 | **QRSexplanation** | In case of QRS width < 150ms, proof of asynchrony |  |  |
| 232 | **AtrialFibrillation** | Presence of atrial fibrillation (AF) during year before impantation | "Y" or "N" (yes or no) |  |
| 233 | **AtrialFibrillationType** | If "Y": type of AF | 30017**01 =**paroxysmal, -**02 =** persistent, **-03 =** permanent" |  |
| 234 | **PacingIndication** | Indication for pacing | "Y"or "N" (yes or no) |  |
| 235 | **LevelPacing** | If "Y": location | 30018**01 =** atrial, -**02 =** ventricular, **-03 =** both |  |
| 236 | **PacemakerCarrier** | Pacemaker carrier at time of ICD implantation | "Y"or "N" (yes or no) |  |
| 237 | **Pacemakerimplantationdate** | If "Y": implantation date of pacemaker |  |  |
| 238 | **Pacemakeridentificationcode** | If "Y": identification code of pacemaker |  |  |
| 239 | **PacemakerFirm** | If "Y": firm of pacemaker |  |  |
| 240 | **PacemakerModel** | If "Y": model of pacemaker |  |  |
| 241 | **Comorbidity-Diabetes** | Presence of diabetes | "Y"or "N" (yes or no) |  |
| 242 | **Comobidity-COPD** | Presence of COPD | "Y"or "N" (yes or no) |  |
| 243 | **Comobidity-Neurological** | Neurological history | "Y"or "N" (yes or no) |  |
| 244 | **Comorbidity-Oncological** | Oncological history | "Y"or "N" (yes or no) |  |
| 245 | **OncologicalExplanation** | Description of oncological history |  |  |
| 246 | **Comorbidity-RenalFailure** | Presence of renal failure | "Y"or "N" (yes or no) |  |
| 247 | **Comorbidity-Creatinine** | Creatinine (mg/dL) |  |  |
| 248 | **RenalFailure-Type** | Type of value on renal failure | 30020**01 =** BUN, **-02 =** urea, **-03 =** BUN and urea |  |
| 249 | **Comorbidity-BUN** | Value of BUN |  |  |
| 250 | **Comorbidity-UREA** | Value of urea |  |  |
| 251 | **Comorbidity-NONE** | No of previous comorbidities | "Y"or "N" (yes or no) |  |
| 252 | **Comorbidity-GFR** | GFR in mL/min/1,73m² |  |  |
| 253 | **CommentsComobidity** | General remarks on comorbidities |  |  |
| 254 | **ResultT-waveAlternans** | Result of T-wave alternans | 30021**01 =** positive, **-02 =** negative, **-03 =** doubtful |  |
| 255 | **ResultBaroreceptorSensitivity** | Result of baroreceptor sensitivity | 30021**01 =** positive, **-02 =** negative, **-03 =** doubtful |  |
| 256 | **ResultHeartRateVariability** | Result of heart rate variability | 30023**01 =** predictive, **-02 =** non predictive, **-03 =** doubtful |  |
| 257 | **ResultHoltermonitoring** | Result of holtermonitoring | 30023**01 =** predictive, **-02 =** non predictive, **-03 =** doubtful |  |
| 258 | **ResultElectrofysiologicalIExam** | Result of electrofysiological exam | 30023**01 =** predictive, **-02 =** non predictive, **-03 =** doubtful |  |
| 259 | **OtherResults** | Other relevant results |  |  |
| 260 | **CommentsCriteria** | General remarks on criteria |  |  |
| 261 | **Registrationcode** | Registration code of implantation |  |  |
| 262 | **OriginalRegistrationcode** | Original registration code of correction was provided |  |  |
| 263 | **StatusRegistration** | Status of registration |  |  |
| 264 | **ChannelType** | Mode of data input | "database import", "web application", "web service" |  |

***Table 1a:*** ***Original data fields registry.*** *Dates formatted as dd/mm/yyyy; * classification changed during period of data collection; CBSS = Crossroads Bank for Social Security; TOF = Tetralogy of Fallot; TGA = Transposition of the Great Arteries; DORV = Double Outlet Right Ventricle; LQTS = Long QT Syndrome; BrS = Brugada syndrome; HCM = Hypetrophic cardiomyopathy; ARVC = Arrhythmogenic Right Ventricle Cardiomyopathy; VF = Ventricular Fibrillation; VT = Ventricular Tachycardia; RV = Right Ventricle; RA = Right Atrium; LV = Left Ventricle*

| **NR** | **DATA FIELD** | **CODING** | **NEW FIELD NAME** | **NEW CODING** |
| --- | --- | --- | --- | --- |
| 92 | **NYHAclass** | 30013**01** = class I, -**02** = class IIa, **-03** = class IIb, -**04** = class III, -**05** = class IV | **NYHA** | I, II, III, IV |
| 105 | **ElectrodeConfiguration** | 30036**01 =** RV, **-02 =** RA+RV, **-03 =** RA+RV+LV, **-04=** RV+LV | **EC** | VVI/DDD or CRT-D |
| 229 | **LBBB** | "Y"or "N" (yes or no) | **LBBB** | 1 or 0 |
| 230 | **QRS duration** | *see table 4* | **QRS** | < 120ms, 120 - 150ms, 150 - 180ms, > 180ms |
| 232 | **AtrialFibrillation** | "Y"or "N" (yes or no) | **AF** | 1 or 0 |
| 233 | **AtrialFibrillationType** | 30017**01 =**paroxysmal, -**02 =** persistent, **-03 =** permanent" | **AF_type** | 0, paroxysmal, persistent or permanent |
| 241 | **Comorbidity-Diabetes** | "Y"or "N" (yes or no) | **Diabetes** | 1 or 0 |
| 242 | **Comorbidity-COPD** | "Y"or "N" (yes or no) | **COPD** | 1 or 0 |
| 243 | **Comorbidity-Neurological** | "Y"or "N" (yes or no) | **Neurological** | 1 or 0 |
| 244 | **Comorbidity-Oncological** | "Y"or "N" (yes or no) | **Oncological** | 1 or 0 |
| 246 | **Comorbidity-RenalFailure** | "Y"or "N" (yes or no) | **RF** | 1 or 0 |
| 251 | **Comorbidity-NONE** | "Y"or "N" (yes or no) | **None** | 1 or 0 |
| **265** |  |  | **Pathology** | ischemic CMP, non-ischemic CMP, congenital, CMP_ARVC, CMP_Brugada, CMP_LQT, CMP_HCM, CMP_Sarcoidosis, CMP_otherarrhythmogenic, miscellaneous |
| **266** |  |  | **Prevention** | primary, secundary - syncope, secundary - reanimation |
| **267** |  |  | **Heart Disease** | Arrhythmogenic CMP, Congenital CMP, ischemic CMP, non-ischemic CMP, other CMP |
| **268** |  |  | **NIS code** | *NIS code based on data field 'District'* |
| **269** |  |  | **Arrondissement** | area of residency based on *'NIS code'* |
| **270** |  |  | **Center volume** | High or low |
| **271** |  |  | **Category Population** | High or middle or low |
| **272** |  |  | **Category Income** | High or middle or low |

***Table 1b****:* ***Adjusted data fields.***  *Presentation of the original data field with description (left columns) and the recoded data field with description (right columns). For new data fields, the left columns are left blank.*

***Table 2:*** ***Type of prevention.*** *Coding system of the health care institution for the data field ‘ConventionalIndication’. * Additional information necessary to define primary or secondary prevention. NA = not applicable as implantations during this timeframe were excluded.*

***Table 3: Symptoms***

***Table 4***: ***QRS duration***

***Table 5: Aetiology***

***Table 6: Classification to underlying cardiomyopathy***

| **INDICATION** | **AETIOLOGY** | **AFFECTION** | **PATHOLOGY** |
| --- | --- | --- | --- |
| 1 or 2 or 3.1 or 4 | C3 until C10 |  | **ICMP** |
| 4.1 or 7.1 or 7.2 |  |  |  |
| 1 or 2 or 3.1 or 4 | C11 until C14 |  | **NICM** |
| 1 or 2 or 3.1 or 4 | G3 or G4 |  |  |
| 4.2 or 7.3 or 7.4 |  |  |  |
| 1 or 2 or 3.1 or 4 | H2 |  | **CMP_LQT** |
| 1 or 2 or 3.1 or 4 |  | 5.1 |  |
| 4.3 |  |  |  |
| 1 or 2 or 3.1 or 4 |  | 5.2 | **CMP_Brugada** |
| 4.4 |  |  |  |
| 1 or 2 or 3.1 or 4 |  | 5.5 | **CMP_other_ar** |
| 4.5 |  |  |  |
| 1 or 2 or 3.1 or 4 | G5 |  | **CMP_HCM** |
| 1 or 2 or 3.1 or 4 |  | 5.3 |  |
| 4.6 |  |  |  |
| 1 or 2 or 3.1 or 4 | G6 |  | **CMP_ARVC** |
| 1 or 2 or 3.1 or 4 |  | 5.4 |  |
| 4.7 |  |  |  |
| 1 or 2 or 3.1 or 4 |  | Congenital | **Congenital** |
| 6 |  |  | **CMP_sarcoidosis** |

# II Additional statistical analyses

## Sex differences stratified by center volume

|  |  | **High Volume** | |  |  |  | **Low Volume** | |  |  |  |
| --- | --- | --- | --- | --- | --- | --- | --- | --- | --- | --- | --- |
|  |  | **Men** | **%** | **Women** | **%** | **p-value** | **Men** | **%** | **Women** | **%** | **p-value** |
|  |  |  |  |  |  |  |  |  |  |  |  |
| N= |  | 7643 | 78.9 | 2045 | 21.1 |  | 4048 | 79.4 | 1051 | 20.6 |  |
|  |  |  |  |  |  |  |  |  |  |  |  |
| **Age** |  | Mean | SD | Mean | SD | <0.001 | Mean | SD | Mean | SD | <0.001 |
|  |  | 62.9 | 13.3 | 59.9 | 15.3 |  | 62.0 | 12.8 | 58.9 | 14.7 |  |
| **LVEF** |  | Mean | SD | Mean | SD | <0.001 | Mean | SD | Mean | SD | <0.001 |
|  |  | 34.9 | 15.1 | 37.8 | 17.4 |  | 33.5 | 15.1 | 36.1 | 15.5 |  |
| **NYHA** | I | 1124 | 14.7% | 434.0 | 21.2% | <0.001 | 573 | 14.2% | 193 | 18.4% | <0.001 |
|  | II | 4289 | 56.1% | 896.0 | 43.8% |  | 2420 | 59.8% | 527 | 50.1% |  |
|  | III | 2217 | 29.0% | 711.0 | 34.8% |  | 1047 | 25.9% | 329 | 31.3% |  |
|  | IV | 13 | 0.2% | 4.0 | 0.2% |  | 8 | 0.2% | 2 | 0.2% |  |
| **Prevention** | Primary | 5125 | 67.1% | 1316.0 | 64.4% | 0.021 | 2895 | 71.5% | 723 | 68.8% | 0.083 |
|  | Secondary | 2518 | 32.9% | 729.0 | 35.6% |  | 1153 | 28.5% | 328 | 31.2% |  |
| **Heart Disease** | IHD | 4101 | 53.7% | 548.0 | 26.8% | <0.001 | 2231 | 55.1% | 317 | 30.2% | <0.001 |
|  | NIHD | 2345 | 30.7% | 918.0 | 44.9% |  | 1261 | 31.2% | 468 | 44.5% |  |
|  | AHD | 1055 | 13.8% | 497.0 | 24.3% |  | 460 | 11.4% | 217 | 20.6% |  |
|  | ACHD | 20 | 0.3% | 13.0 | 0.6% |  | 11 | 0.3% | 5 | 0.5% |  |
|  | Other | 122 | 1.6% | 69.0 | 3.4% |  | 85 | 2.1% | 44 | 4.2% |  |
| **Type Device** | VVI/DDD | 5606 | 73.3% | 1372.0 | 67.1% | <0.001 | 3153 | 77.9% | 743 | 70.7% | <0.001 |
|  | CRT-D | 2037 | 26.7% | 673.0 | 32.9% |  | 895 | 22.1% | 308 | 29.3% |  |
| **QRS** | <120 | 5098 | 66.7% | 1261.0 | 61.7% | <0.001 | 2701 | 66.7% | 644 | 61.3% | 0.002 |
|  | 120-150 | 653 | 8.5% | 169.0 | 8.3% |  | 390 | 9.6% | 113 | 10.8% |  |
|  | 150-180 | 1433 | 18.7% | 530.0 | 25.9% |  | 722 | 17.8% | 237 | 22.5% |  |
|  | >180 | 459 | 6.0% | 85.0 | 4.2% |  | 235 | 5.8% | 57 | 5.4% |  |
| **Complications** | <365 days | 170 | 2.2% | 75.0 | 3.7% | <0.001 | 142 | 3.5% | 57 | 5.4% | 0.004 |
| **AF** |  | 1841 | 24.1% | 369.0 | 18.0% | <0.001 | 936 | 23.1% | 178 | 16.9% | <0.001 |
| **Diabetes** |  | 992 | 13.0% | 217.0 | 10.6% | 0.004 | 838 | 20.7% | 162 | 15.4% | <0.001 |
| **COPD** |  | 469 | 6.1% | 88.0 | 4.3% | 0.002 | 410 | 10.1% | 85 | 8.1% | 0.046 |
| **Neurological** |  | 381 | 5.0% | 70.0 | 3.4% | 0.003 | 284 | 7.0% | 59 | 5.6% | 0.106 |
| **Oncological** |  | 212 | 2.8% | 123.0 | 6.0% | <0.001 | 148 | 3.7% | 69 | 6.6% | <0.001 |
| **Renal Failure** |  | 692 | 9.1% | 154.0 | 7.5% | 0.030 | 533 | 13.2% | 96 | 9.1% | <0.001 |
| **Population** | high | 3568 | 46.7% | 981.0 | 48.0% | 0.391 | 1848 | 45.7% | 508 | 48.3% | 0.293 |
| **Density** | middle | 4001 | 52.3% | 1049.0 | 51.3% |  | 1420 | 35.1% | 348 | 33.1% |  |
|  | low | 74 | 1.0% | 15.0 | 0.7% |  | 780 | 19.3% | 195 | 18.6% |  |
| **Income** | high | 2956 | 38.7% | 799.0 | 39.1% | 0.747 | 728 | 18.0% | 187 | 17.8% | 0.693 |
|  | middle | 634 | 8.3% | 178.0 | 8.7% |  | 2111 | 52.1% | 563 | 53.6% |  |
|  | low | 4053 | 53.0% | 1068.0 | 52.2% |  | 1209 | 29.9% | 301 | 28.6% |  |

Table 1. Baseline patient characteristics stratified by center volume with comparison of sex. Continuous variables expressed as mean ± SD and categorical variables as number with %. LVEF = Left Ventricular Ejection Fraction, NYHA = New York Heart Association classification of heart failure, AF = Atrial Fibrillation, COPD = Chronic Obstructive Pulmonary Disease. Population Density and Income divided in low (percentile 0-25), middle (percentile 25-75) and high (percentile 75-100).

## Center volume differences stratified by sex

|  |  | **Women** |  |  |  |  | **Men** |  |  |  |  |
| --- | --- | --- | --- | --- | --- | --- | --- | --- | --- | --- | --- |
|  |  | **High** | **%** | **Low** | **%** | **p-value** | **High** | **%** | **Low** | **%** | **p-value** |
|  |  |  |  |  |  |  |  |  |  |  |  |
| N= |  | 2045 | 66.1% | 1051 | 33.9% |  | 7643 | 65.4% | 4048 | 34.6% |  |
|  |  |  |  |  |  |  |  |  |  |  |  |
| **Age** |  | Mean | SD | Mean | SD | 0.008 | Mean | SD | Mean | SD | <0.001 |
|  |  | 59.9 | 15.3 | 58.9 | 14.7 |  | 62.9 | 13.3 | 62.0 | 12.8 |  |
| **LVEF** |  | Mean | SD | Mean | SD | 0.043 | Mean | SD | Mean | SD | <0.001 |
|  |  | 37.8 | 17.4 | 36.1 | 15.8 |  | 34.9 | 15.1 | 33.5 | 13.7 |  |
| **NYHA** | I | 434 | 21.2% | 193.0 | 18.4% | 0.010 | 1124 | 14.7% | 573 | 14.2% | 0.001 |
|  | II | 896 | 43.8% | 527.0 | 50.1% |  | 4289 | 56.1% | 2420 | 59.8% |  |
|  | III | 711 | 34.8% | 329.0 | 31.3% |  | 2217 | 29.0% | 1047 | 25.9% |  |
|  | IV | 4 | 0.2% | 2.0 | 0.2% |  | 13 | 0.2% | 8 | 0.2% |  |
| **Prevention** | Primary | 1316 | 64.4% | 723.0 | 68.8% | 0.014 | 5125 | 67.1% | 2895 | 71.5% | <0.001 |
|  | Secondary | 729 | 35.6% | 328.0 | 31.2% |  | 2518 | 32.9% | 1153 | 28.5% |  |
| **Heart Disease** | IHD | 548 | 26.8% | 317.0 | 30.2% | 0.077 | 4101 | 53.7% | 2231 | 55.1% | 0.002 |
|  | NIHD | 918 | 44.9% | 468.0 | 44.5% |  | 2345 | 30.7% | 1261 | 31.2% |  |
|  | AHD | 497 | 24.3% | 217.0 | 20.6% |  | 1055 | 13.8% | 460 | 11.4% |  |
|  | ACHD | 13 | 0.6% | 5.0 | 0.5% |  | 20 | 0.3% | 11 | 0.3% |  |
|  | Other | 69 | 3.4% | 44.0 | 4.2% |  | 122 | 1.6% | 85 | 2.1% |  |
| **Type Device** | VVI/DDD | 1372 | 67.1% | 743.0 | 70.7% | 0.041 | 5606 | 73.3% | 3153 | 77.9% | <0.001 |
|  | CRT-D | 673 | 32.9% | 308.0 | 29.3% |  | 2037 | 26.7% | 895 | 22.1% |  |
| **QRS** | <120 | 1261 | 61.7% | 644.0 | 61.3% | 0.016 | 5098 | 66.7% | 2701 | 66.7% | 0.179 |
|  | 120-150 | 169 | 8.3% | 113.0 | 10.8% |  | 653 | 8.5% | 390 | 9.6% |  |
|  | 150-180 | 530 | 25.9% | 237.0 | 22.5% |  | 1433 | 18.7% | 722 | 17.8% |  |
|  | >180 | 85 | 4.2% | 57.0 | 5.4% |  | 459 | 6.0% | 235 | 5.8% |  |
| **Complications** | <365 days | 75 | 3.7% | 57.0 | 5.4% | 0.022 | 170 | 2.2% | 142 | 3.5% | <0.001 |
| **AF** |  | 369 | 18.0% | 178.0 | 16.9% | 0.444 | 1841 | 24.1% | 936 | 23.1% | 0.243 |
| **Diabetes** |  | 217 | 10.6% | 162.0 | 15.4% | <0.001 | 992 | 13.0% | 838 | 20.7% | <0.001 |
| **COPD** |  | 88 | 4.3% | 85.0 | 8.1% | <0.001 | 469 | 6.1% | 410 | 10.1% | <0.001 |
| **Neurological** |  | 70 | 3.4% | 59.0 | 5.6% | 0.004 | 381 | 5.0% | 284 | 7.0% | <0.001 |
| **Oncological** |  | 123 | 6.0% | 69.0 | 6.6% | 0.548 | 212 | 2.8% | 148 | 3.7% | 0.009 |
| **Renal Failure** |  | 154 | 7.5% | 96.0 | 9.1% | 0.121 | 692 | 9.1% | 533 | 13.2% | <0.001 |
| **Population Density** | high | 981 | 48.0% | 508.0 | 48.3% | <0.001 | 3568 | 46.7% | 1848 | 45.7% | <0.001 |
|  | middle | 1049 | 51.3% | 348.0 | 33.1% |  | 4001 | 52.3% | 1420 | 35.1% |  |
|  | low | 15 | 0.7% | 195.0 | 18.6% |  | 74 | 1.0% | 780 | 19.3% |  |
| **Income** | high | 799 | 39.1% | 187.0 | 17.8% | <0.001 | 2956 | 38.7% | 728 | 18.0% | <0.001 |
|  | middle | 1068 | 52.2% | 301.0 | 28.6% |  | 4053 | 53.0% | 1209 | 29.9% |  |
|  | low | 178 | 8.7% | 563.0 | 53.6% |  | 634 | 8.3% | 2111 | 52.1% |  |

Table 2. Baseline patient characteristics stratified by sex with comparison of center volume. Continuous variables expressed as mean ± SD and categorical variables as number with %. LVEF = Left Ventricular Ejection Fraction, NYHA = New York Heart Association classification of heart failure, AF = Atrial Fibrillation, COPD = Chronic Obstructive Pulmonary Disease. Population Density and Income divided in low (percentile 0-25), middle (percentile 25-75) and high (percentile 75-100).

## Interaction term of sex and center volume

To check for discrepancies in sex-specific differences according to center volume, we introduced an interaction term for sex and center volume (sex*centervolume). A binary or ordinal generalized linear model was built as appropriate, to test the interaction term for categorical variables. Linear regression was used to test for age and LVEF.

|  |  | **Sex** | **Center Volume** | **Sex*Center Volume** |
| --- | --- | --- | --- | --- |
|  |  |  |  |  |
| **Age** |  | <0,001 | <0,001 | 0.979 |
| **LVEF** |  | <0,001 | <0,001 | 0.640 |
| **NYHA** |  | <0,001 | 0.003 | 0.757 |
| **Prevention** |  | <0,001 | <0,001 | 0.911 |
| **Heart Disease** |  | <0,001 | 0.175 | 0.051 |
| **Type Device** |  | <0,001 | <0,001 | 0.405 |
| **QRS** |  | <0,001 | 0.769 | 0.812 |
| **Complications** | <365 days | <0,001 | <0,001 | 0.782 |
| **AF** |  | <0,001 | 0.244 | 0.833 |
| **Diabetes** |  | <0,001 | <0,001 | 0.286 |
| **COPD** |  | <0,001 | <0,001 | 0.462 |
| **Neurological** |  | <0,001 | <0,001 | 0.436 |
| **Oncological** |  | <0,001 | 0.009 | 0.312 |
| **Renal Failure** |  | <0,001 | <0,001 | 0.159 |
| **Population Density** |  | 0.332 | <0,001 | 0.436 |
| **Income** |  | 0.723 | <0,001 | 0.359 |

Table 3. Overview of significance levels of generalized linear models to check for interaction between sex and center volume (sex*centervolume).

## Complication rates

|  |  | **Total** | **%** | **Men** | **%** | **Women** | **%** | **p-value** |
| --- | --- | --- | --- | --- | --- | --- | --- | --- |
| **Reinterventions** | <1 y | 444 | 3.00 | 312 | 2.67 | 132 | 4.26 | **<0.001** |
| Dislocation |  | 110 | 0.74 | 64 | 0.55 | 46 | 1.49 | **<0.001** |
| Perforation |  | 12 | 0.08 | 5 | 0.04 | 7 | 0.23 | **0.001** |
| Infection |  | 88 | 0.60 | 72 | 0.62 | 16 | 0.52 | 0.524 |
| Diaphragm stimulation |  | 4 | 0.03 | 3 | 0.03 | 1 | 0.03 | 0.842 |
| Lead problem |  | 110 | 0.74 | 86 | 0.74 | 24 | 0.78 | 0.820 |
| Device failure |  | 10 | 0.07 | 9 | 0.08 | 1 | 0.03 | 0.395 |
| Unspecified |  | 110 | 0.74 | 73 | 0.62 | 37 | 1.20 | **0.001** |

Table 4. Complications within 1 year after primo implantation with need of additional implanted materials. The category ‘lead problem’ consists of lead fractures, conductor breaks, insulation breaks, pacing exit blocks and sensing problems. The category ‘device failure’ consists of recalls, premature battery depletions, generator problems without further specification and necessity for transvenous ICD device after failure of subcutaneous ICD device. Interventions without clear description are classified as ‘unspecified’.

## Univariable cox regression

|  |  |  | ALL (N=14787) | | | | | MEN (N=11691) | | | | WOMEN (N=3096) | | | |
| --- | --- | --- | --- | --- | --- | --- | --- | --- | --- | --- | --- | --- | --- | --- | --- |
|  |  |  | HR | *95% CI* |  | p-value | HR | | *95% CI* |  | p-value | HR | *95% CI* |  | p-value |
|  |  |  |  |  |  |  |  | |  |  |  |  |  |  |  |
| **Sex** | F |  |  |  |  |  |  | |  |  |  |  |  |  |  |
|  | M |  | 1.358 | *1.218* | *1.514* | <0.001 |  | |  |  |  |  |  |  |  |
| **Age** |  |  | 1.059 | *1.055* | *1.064* | <0.001 | 1.058 | | *1.054* | *1.063* | <0.001 | 1.062 | *1.051* | *1.072* | <0.001 |
| **LVEF** |  |  | 0.976 | *0.973* | *0.979* | <0.001 | 0.976 | | *0.973* | *0.980* | <0.001 | 0.976 | *0.969* | *0.983* | <0.001 |
| **NYHA** | I |  |  |  |  |  |  | |  |  |  |  |  |  |  |
|  | II |  | 2.886 | *2.438* | *3.415* | <0.001 | 2.634 | | *2.191* | *3.166* | <0.001 | 3.886 | *2.544* | *5.873* | <0.001 |
|  | III |  | 3.839 | *3.234* | *4.556* | <0.001 | 3.662 | | *3.035* | *4.418* | <0.001 | 4.624 | *3.042* | *7.030* | <0.001 |
|  | IV |  | 7.458 | *3.933* | *14.143* | <0.001 | 6.071 | | *2.971* | *12.404* | <0.001 | 17.693 | *4.182* | *74.861* | <0.001 |
| **Prevention** | Primary |  |  |  |  |  |  | |  |  |  |  |  |  |  |
|  | Secondary |  | 1.085 | *0.998* | *1.179* | 0.056 | 1.093 | | *0.998* | *1.197* | 0.056 | 1.083 | *0.882* | *1.328* | 0.447 |
| **Heart Disease** | Arrhythmogenic |  |  |  |  |  |  | |  |  |  |  |  |  |  |
|  | Ischemic |  | 3.508 | *2.955* | *4.164* | <0.001 | 3.423 | | *2.798* | *4.189* | <0.001 | 3.679 | *2.621* | *5.163* | <0.001 |
|  | Non-ischemic |  | 2.667 | *2.232* | *3.186* | <0.001 | 2.805 | | *2.274* | *3.460* | <0.001 | 2.246 | *1.603* | *3.149* | <0.001 |
|  | Congenital |  | 1.665 | *0.780* | *3.556* | 0.188 | 1.774 | | *0.723* | *4.355* | 0.211 | 1.464 | *0.354* | *6.047* | 0.599 |
|  | Other |  | 1.298 | *0.829* | *2.033* | 0.255 | 1.331 | | *0.774* | *2.288* | 0.302 | 1.241 | *0.558* | *2.763* | 0.588 |
| **Type of Device** | VVI/DDD |  |  |  |  |  |  | |  |  |  |  |  |  |  |
|  | CRT-D |  | 1.304 | *1.195* | *1.424* | <0.001 | 1.358 | | *1.233* | *1.495* | <0.001 | 1.191 | *0.965* | *1.469* | 0.104 |
| **QRS (ms)** | <120 |  |  |  |  |  |  | |  |  |  |  |  |  |  |
|  | 120-150 |  | 1.580 | *1.372* | *1.820* | <0.001 | 1.573 | | *1.346* | *1.838* | <0.001 | 1.701 | *0.975* | *2.346* | 0.065 |
|  | 150-180 |  | 1.436 | *1.301* | *1.584* | <0.001 | 1.502 | | *1.347* | *1.675* | <0.001 | 1.354 | *1.074* | *1.706* | 0.010 |
|  | >180 |  | 1.346 | *1.140* | *1.589* | <0.001 | 1.310 | | *1.095* | *1.568* | 0.003 | 1.513 | *0.975* | *2.346* | 0.065 |
| **AF** |  |  | 1.978 | *1.816* | *2.154* | <0.001 | 1.924 | | *1.753* | *2.111* | <0.001 | 2.130 | *1.709* | *2.654* | <0.001 |
| **Diabetes** |  |  | 1.781 | *1.613* | *1.967* | <0.001 | 1.631 | | *1.462* | *1.819* | <0.001 | 2.645 | *2.078* | *3.365* | <0.001 |
| **COPD** |  |  | 2.043 | *1.804* | *2.314* | <0.001 | 2.038 | | *1.783* | *2.329* | <0.001 | 1.921 | *1.363* | *2.707* | <0.001 |
| **Neurological** |  |  | 1.532 | *1.315* | *1.785* | <0.001 | 1.436 | | *1.216* | *1.696* | <0.001 | 2.036 | *1.386* | *2.991* | <0.001 |
| **Oncological** |  |  | 1.540 | *1.283* | *1.847* | <0.001 | 1.707 | | *1.388* | *2.100* | <0.001 | 1.321 | *0.899* | *1.940* | 0.156 |
| **Renal failure** |  |  | 2.480 | *2.223* | *2.766* | <0.001 | 2.315 | | *2.055* | *2.607* | <0.001 | 3.369 | *2.558* | *4.439* | <0.001 |
| **Complications** |  |  | 0.936 | *0.726* | *1.207* | 0.612 | 0.904 | | *0.670* | *1.219* | 0.507 | 1.142 | *0.702* | *1.858* | 0.592 |
| **Center Volume** | high |  |  |  |  |  |  | |  |  |  |  |  |  |  |
|  | low |  | 1.287 | *1.185* | *1.398* | <0.001 | 1.302 | | *1.190* | *1.425* | <0.001 | 1.210 | *0.984* | *1.489* | 0.070 |
| **Population density** | high |  |  |  |  |  |  | |  |  |  |  |  |  |  |
|  | middle |  | 0.887 | *0.816* | *0.964* | 0.005 | 0.886 | | *0.809* | *0.971* | 0.010 | 0.868 | *0.706* | *1.068* | 0.180 |
|  | low |  | 0.986 | *0.842* | *1.154* | 0.859 | 1.018 | | *0.859* | *1.208* | 0.834 | 0.711 | *0.531* | *1.238* | 0.331 |
| **Income** | high |  |  |  |  |  |  | |  |  |  |  |  |  |  |
|  | middle |  | 0.949 | *0.862* | *1.044* | 0.281 | 0.967 | | *0.871* | *1.073* | 0.525 | 0.861 | *0.675* | *1.098* | 0.227 |
|  | low |  | 1.324 | *1.192* | *1.471* | <0.001 | 1.293 | | *1.152* | *1.451* | <0.001 | 1.491 | *1.158* | *1.919* | 0.002 |

Table 5. Presentation of unadjusted hazard ratios (HR) with their 95% confidence interval and level of significance (p-value) for the whole group and stratified by sex category.

## Propensity score matching

### Propensity score matching without replacement

Propensity score matching was performed using the nearest neighbor method without replacement for the patients with ischemic heart disease (IHD), non-ischemic heart disease (NIHD) and arrhythmogenic heart disease (AHD), using common support and a caliper set at 0.0005. Patients were matched for age, LVEF, NYHA functional status, type of prevention, type of heart disease, type of device, QRS width, presence of atrial fibrillation (AF), diabetes mellitus and renal failure.

|  |  | **Total** | **%** | **Men** | **%** | **Women** | **%** | **p-value** |
| --- | --- | --- | --- | --- | --- | --- | --- | --- |
|  |  |  |  |  |  |  |  |  |
| N= |  | 5468 | 100 | 2734 | 50 | 2734 | 50 |  |
| **Age** |  | Mean | SD | Mean | SD | Mean | SD |  |
|  |  | 59.8 | 14.7 | 59.9 | 14.6 | 59.8 | 14.8 | 0.827 |
| **LVEF** |  | Mean | SD | Mean | SD | Mean | SD |  |
|  |  | 36.3 | 16.4 | 36.4 | 16.6 | 36.2 | 16.2 | 0.730 |
| **NYHA** | I | 1056 | 19.3 | 529 | 19.4 | 527 | 19.3 | 0.492 |
|  | II | 2558 | 46.8 | 1301 | 47.6 | 1257 | 46.0 |  |
|  | III | 1844 | 33.7 | 898 | 32.9 | 946 | 34.6 |  |
|  | IV | 10 | 0.2 | 6 | 0.2 | 4 | 0.2 |  |
| **Prevention** | Primary | 3717 | 68.0 | 1862 | 68.1 | 1855 | 67.9 | 0.839 |
|  | Secondary | 1751 | 32.0 | 872 | 31.9 | 879 | 32.1 |  |
| **Heart Disease** | IHD | 1675 | 30.6 | 851 | 31.1 | 824 | 30.1 | 0.412 |
|  | NIHD | 2467 | 45.1 | 1209 | 44.2 | 1258 | 46.0 |  |
|  | AHD | 1326 | 24.3 | 674 | 24.7 | 652 | 23.9 |  |
| **Type Device** | VVI/DDD | 3702 | 67.7 | 1862 | 68.1 | 1840 | 67.3 | 0.525 |
|  | CRT-D | 1766 | 32.3 | 872 | 31.9 | 894 | 32.7 |  |
| **QRS** | <120 | 3360 | 61.5 | 1696 | 62.0 | 1664 | 60.9 | 0.532 |
|  | 120-150 | 463 | 8.5 | 224 | 8.2 | 239 | 8.7 |  |
|  | 150-180 | 1367 | 25.0 | 668 | 24.4 | 699 | 25.6 |  |
|  | >180 | 278 | 5.1 | 146 | 5.3 | 132 | 4.8 |  |
| **AF** |  | 984 | 18.0 | 491 | 18.0 | 493 | 18.0 | 0.944 |
| **Diabetes** |  | 678 | 12.4 | 323 | 11.8 | 355 | 13.0 | 0.189 |
| **COPD** |  | 266 | 4.9 | 133 | 4.9 | 133 | 4.9 | 1.000 |
| **Neurological** |  | 244 | 4.5 | 133 | 4.9 | 111 | 4.1 | 0.150 |
| **Oncological** |  | 144 | 2.6 | 72 | 2.6 | 72 | 2.6 | 1.000 |
| **Renal Failure** |  | 449 | 8.2 | 228 | 8.3 | 221 | 8.1 | 0.730 |
| **Center Volume** | high | 3632 | 66.4 | 1833 | 67.0 | 1799 | 65.8 | 0.330 |
|  | low | 1836 | 33.6 | 901 | 33.0 | 935 | 34.2 |  |
| **Population Density** | high | 2562 | 46.9 | 1257 | 46.0 | 1305 | 47.7 | 0.429 |
|  | middle | 2529 | 46.2 | 1285 | 47.0 | 1244 | 45.5 |  |
|  | low | 377 | 6.9 | 192 | 7.0 | 185 | 6.8 |  |
| **Income** | high | 1764 | 32.3 | 913 | 33.4 | 851 | 31.1 | 0.146 |
|  | middle | 2442 | 44.7 | 1212 | 44.3 | 1230 | 45.0 |  |
|  | low | 1262 | 23.0 | 609 | 22.3 | 653 | 23.9 |  |
| **Dead** |  | 732 | 13.4 | 388 | 14.2 | 344 | 12.6 | 0.081 |

Table 6. Baseline patient characteristics after propensity score matching. Continuous variables expressed as mean ± SD and categorical variables as number with %. LVEF = Left Ventricular Ejection Fraction, NYHA = New York Heart Association classification of heart failure, AF = Atrial Fibrillation, COPD = Chronic Obstructive Pulmonary Disease. Center Volume with high vs low based on median. Population Density and Income divided in low (percentile 0-25), middle (percentile 25-75) and high (percentile 75-100).

|  |  |  | ALL (N=5468) | | | |
| --- | --- | --- | --- | --- | --- | --- |
|  |  |  | HR | 95% CI |  | p-value |
|  |  |  |  |  |  |  |
| **Sex** | F |  | 0.934 | 0.806 | 1.081 | 0.358 |
| **Age** |  |  | 1.057 | 1.049 | 1.066 | **<0.001** |
| **LVEF** |  |  | 0.985 | 0.978 | 0.993 | **<0.001** |
| **NYHA** | I |  |  |  |  |  |
|  | II |  | 1.364 | 0.957 | 1.943 | 0.086 |
|  | III |  | 2.031 | 1.354 | 3.046 | **0.001** |
|  | IV |  | 2.286 | 0.540 | 9.673 | 0.261 |
| **Prevention** | Primary |  |  |  |  |  |
|  | Secondary |  | 1.374 | 1.164 | 1.621 | **<0.001** |
| **Heart Disease** | Ischemic |  |  |  |  |  |
|  | Non-ischemic |  | 0.842 | 0.713 | 0.993 | **0.042** |
|  | Arrhythmogenic |  | 0.957 | 0.698 | 1.312 | 0.784 |
| **Type of Device** | VVI/DDD |  |  |  |  |  |
|  | CRT-D |  | 0.607 | 0.447 | 0.825 | **0.001** |
| **QRS (ms)** | <120 |  |  |  |  |  |
|  | 120-150 |  | 1.306 | 0.949 | 1.796 | 0.101 |
|  | 150-180 |  | 1.165 | 0.844 | 1.609 | 0.352 |
|  | >180 |  | 0.992 | 0.647 | 1.520 | 0.970 |
| **AF** |  |  | 1.481 | 1.254 | 1.750 | **<0.001** |
| **Diabetes** |  |  | 1.656 | 1.376 | 1.993 | **<0.001** |
| **COPD** |  |  | 1.589 | 1.233 | 2.049 | **<0.001** |
| **Neurological** |  |  | 1.453 | 1.099 | 1.921 | **0.009** |
| **Oncological** |  |  | 1.346 | 0.923 | 1.963 | 0.122 |
| **Renal failure** |  |  | 1.602 | 1.295 | 1.981 | **<0.001** |
| **Center Volume** | high |  | 0.825 | 0.679 | 1.001 | 0.052 |
|  | low |  |  |  |  |  |
| **Population density** | high |  | 1.382 | 0.989 | 1.932 | 0.058 |
|  | middle |  | 1.322 | 0.945 | 1.850 | 0.103 |
|  | low |  |  |  |  |  |
| **Income** | high |  | 0.862 | 0.692 | 1.074 | 0.187 |
|  | middle |  | 0.813 | 0.652 | 1.013 | 0.065 |
|  | low |  |  |  |  |  |

Table 7. Presentation of adjusted hazard ratios (HR) with their 95% confidence interval (95% CI) and level of significance (p-value) after propensity score matching.

### Propensity score matching with multiple imputations

As a sensitivity analysis, additional propensity score matching was performed. The nearest neighbor method with a caliper of 0.1 was used in propensity score matching and standardized mean differences (SMD) were calculated to evaluate adequacy of propensity matching with a threshold of 0.1 (figure 3). In order to estimate the effect of sex on mortality two different propensity score methods were used. In the inverse-probability-weighted analysis with multivariable Cox model, the predicted probabilities from the propensity-score model were used. A second model was conducted using the propensity scores as an additional covariate in the multivariable Cox model (table 4). Missing data was treated by multiple imputations, estimations of imputed datasets were pooled by Rubin`s rule.

|  | HR | 95% CI | p-value |
| --- | --- | --- | --- |
| **Propensity Score Analysis** |  |  |  |
| Adjusted for Propensity Score* | 1.212 | 1.002 1.256 | 0.049 |
| With inverse probability weighting** | 1.108 | 0.927 1.184 | 0.453 |

Table 8. Presentation of hazard ratios (HR) with their 95% confidence interval (95% CI) and level of significance (p-value). * Multivariable Cox proportional-hazards model with additional adjustment for the propensity score. ** Multivariable Cox proportional-hazards model with inverse probability weighting according to the propensity score.


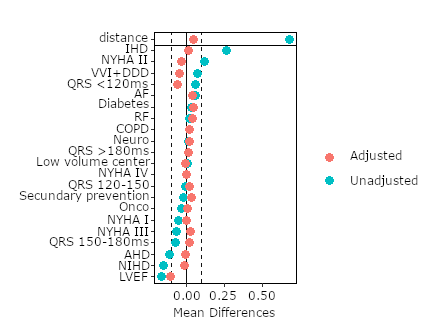


Figure 1. Covariate Balance after Propensity Score Matching

## Survival analysis

###
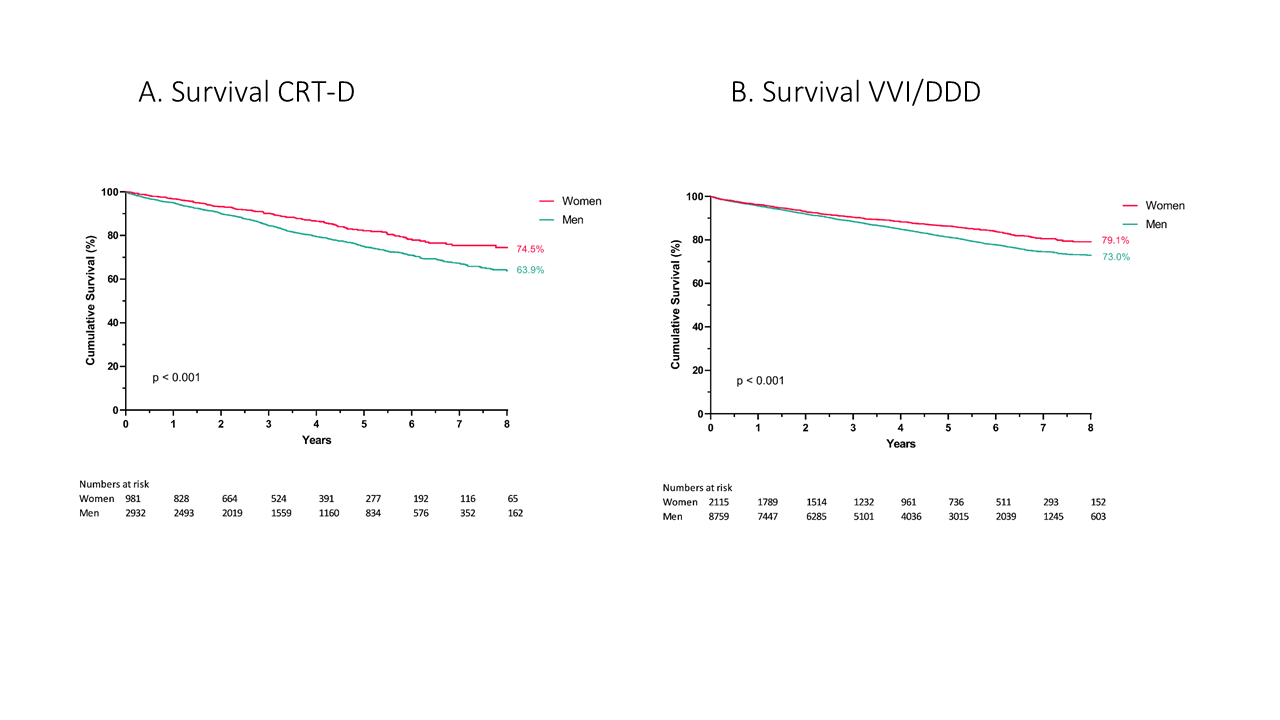
Survival analysis subgroups

Figure 2. Kaplan Meier survival curves by type of device. (A) CRT-D implanted patients, stratified by sex category. Log-rank p < 0.001. (B) VVI/DDD implanted patients, stratified by sex category. Log-rank p < 0.001.


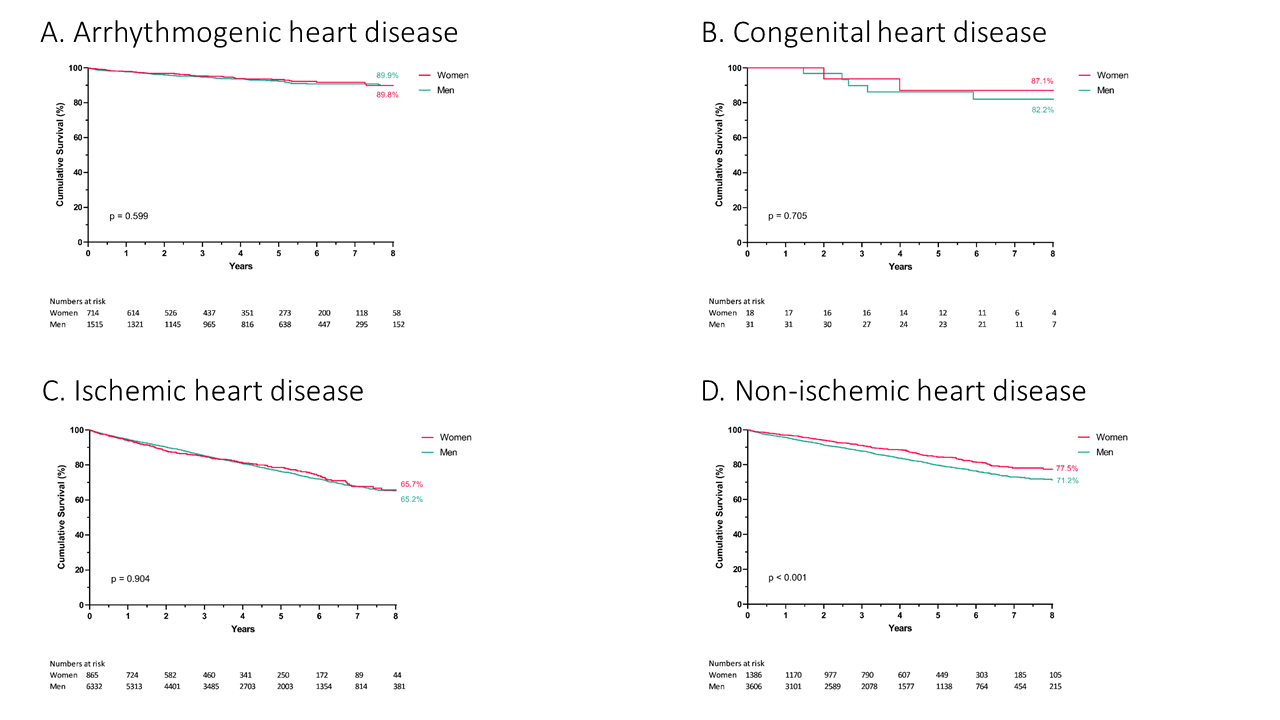


Figure 3. Kaplan Meier survival curves by underlying heart disease. (A) Arrhythmogenic heart disease, stratified by sex category. Log-rank p = 0.599. (B) Congenital heart disease, stratified by sex category. Log-rank p = 0.705. (C) Ischemic heart disease, stratified by sex category. Log-rank p = 0.904. (D) Non-ischemic heart disease, stratified by sex category. Log-rank p <0.001

### Survival analysis after propensity score matching


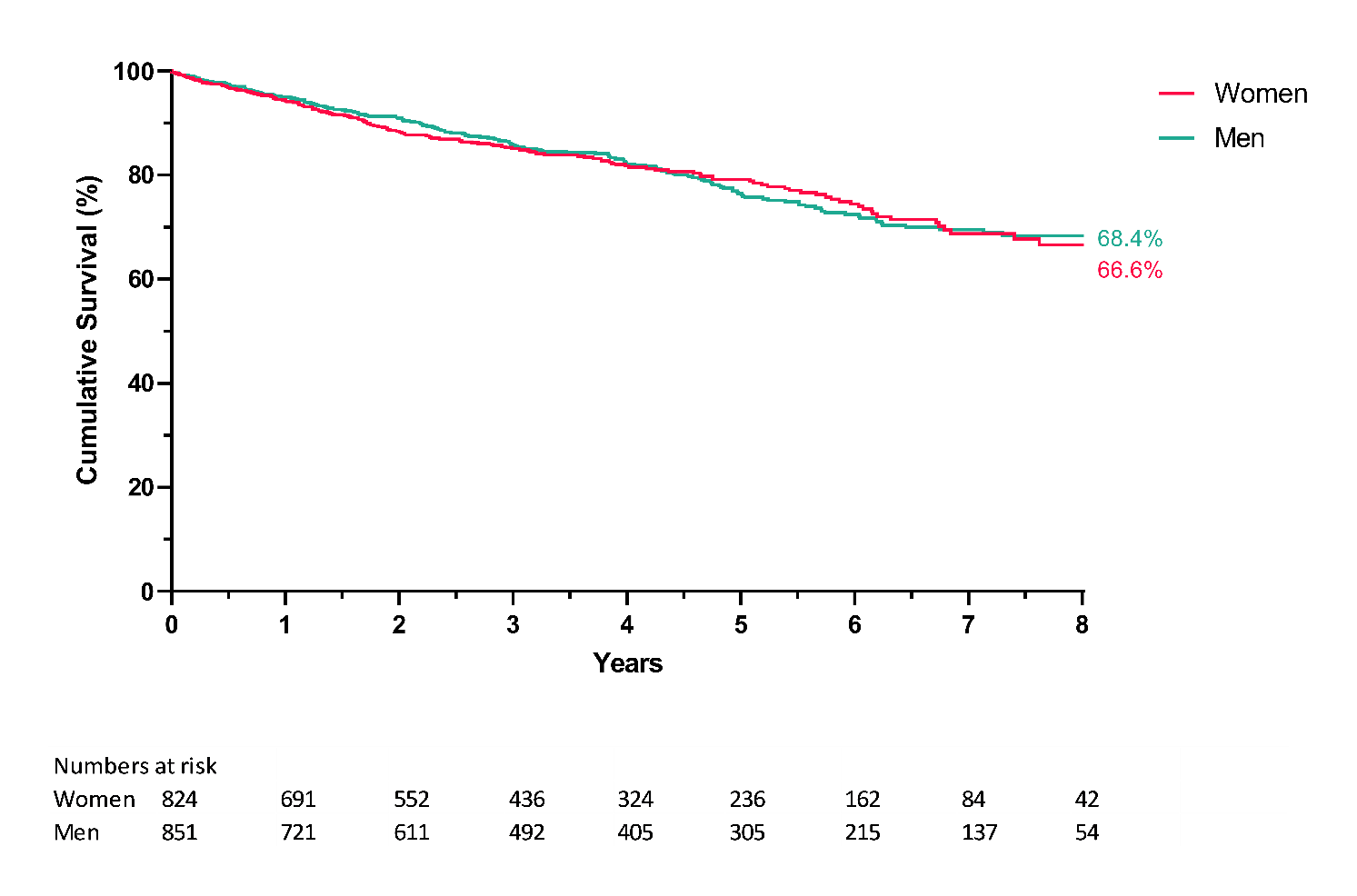


Figure 4. Kaplan Meier survival curve after propensity score matching for IHD patients. Stratification by sex category.


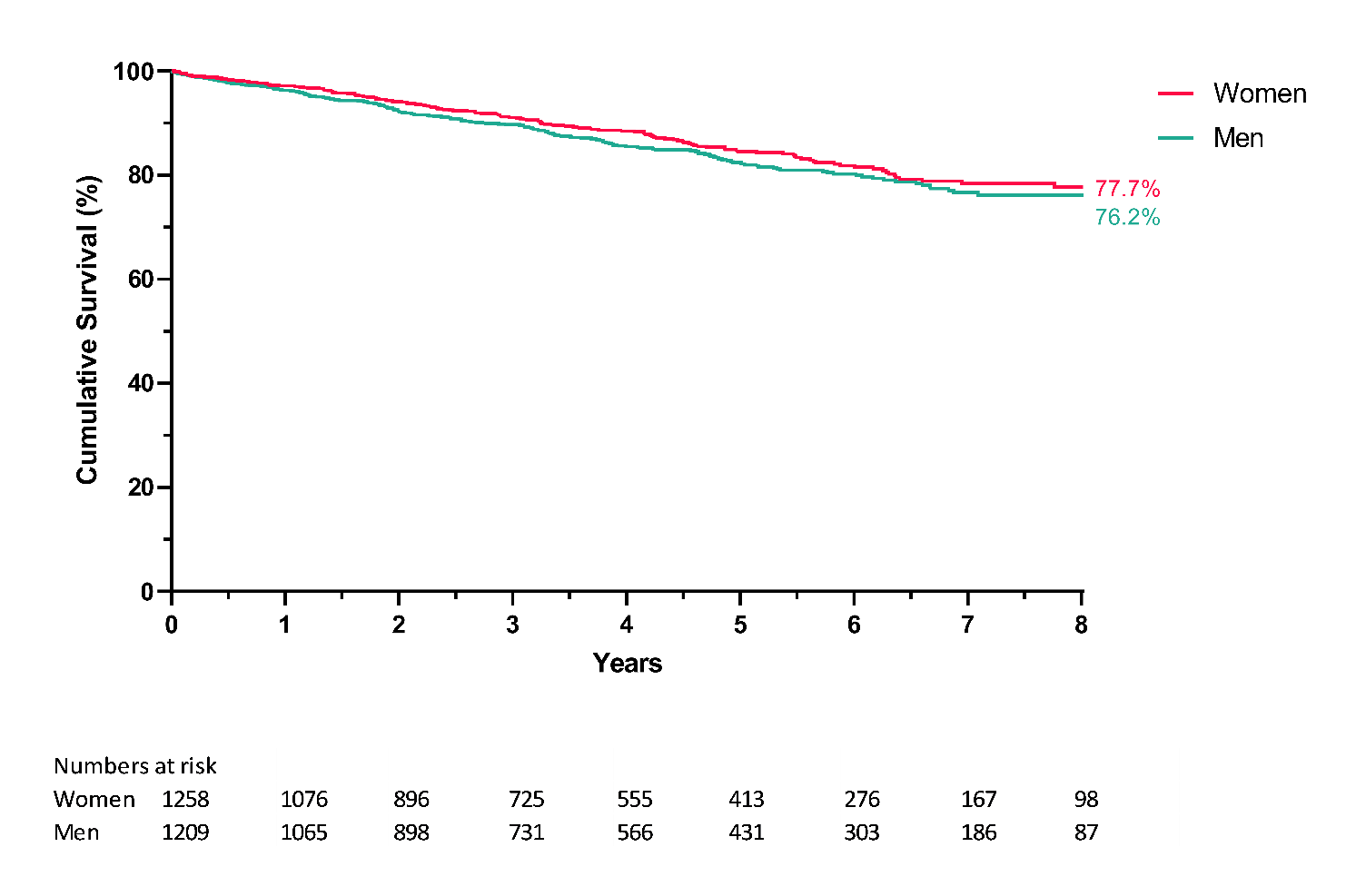


Figure 5. Kaplan Meier survival curve after propensity score matching for NIHD patients. Stratification by sex category.
